# Sesquiterpenoids from *Artemisia vestita* and Their Antifeedant and Antifungal Activities

**DOI:** 10.3390/molecules24203671

**Published:** 2019-10-11

**Authors:** Ying-Hui Ding, Hui-Ting Wang, Sha Shi, Yao Meng, Jin-Chao Feng, Hai-Bo Wu

**Affiliations:** 1 College of Life and Environmental Sciences, Minzu University of China, Beijing 100081, China; dingyinghui1995@163.com (Y.-H.D.); 18801379785@163.com (H.-T.W.); mymengyao95@163.com (Y.M.); fengjinchao@muc.edu.cn (J.-C.F.); ksbb0833279@163.com (H.-B.W.); 2 Center on Translational Neuroscience, Minzu University of China, Beijing 100081, China

**Keywords:** *Artemisia vestita*, sesquiterpenoids, absolute configuration, antifeedant, antifungal

## Abstract

Four new sesquiterpenoids, named artemivestinolide D–G (**1**–**4**) and three known sesquiterpenoids (**5**–**7**), were isolated from *Artemisia vestita.* The structures of these new compounds were determined based on extensive spectroscopic data analyses. Furthermore, the electronic circular dichroism data determined the absolute configurations of the new compounds. The antifeedant and antifungal activities of the isolates were evaluated against third-instar larvae of *Plutella xylostella* and three plant pathogenic fungi. Compounds **1**–**7** showed moderate antifeedant activities and compounds **1**–**4** and **6**–**7** exhibited antifungal activities.

## 1. Introduction

**Figure 1:**
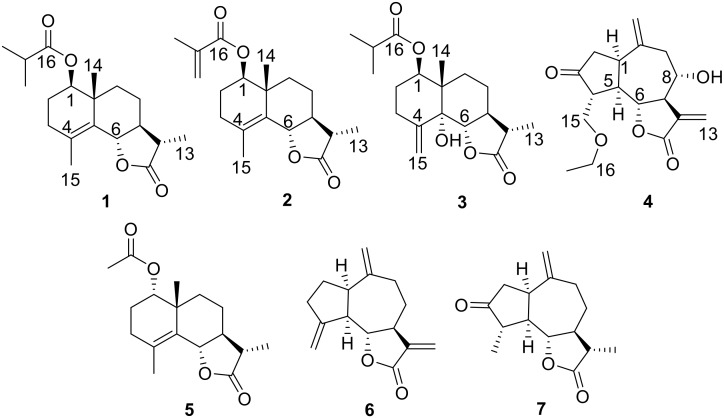
Structures of sesquiterpenoids **1**–**7** from *Artemisia vestita*.

*Artemisia* (Compositae) species are widespread throughout China and receive much attention due to their remarkable biological activities and structural diversities [1,2,3]. *A. vestita* is distributed at wasteland and river beaches of China and it has been widely used in traditional Tibetan and Chinese Traditional Medicine for treating various inflammatory diseases [4]. Modern pharmacological studies indicated that ethanol extracts of *A. vestita* exhibited anti-inflammatory activities [5]. The isolated compounds from *A. vestita* showed inhibitory effects against NO production induced by LPS [6,7]. Furthermore, *A. vestita* was found to possess strong insecticidal activity against *Sitophilus zeamais* and *Haemonchus contortus* [8,9]. A number of sesquiterpenoids have been isolated from *A. vestita* and have shown biological activities [4,5,6]. Sesquiterpenoids have important roles in plants’ defense against pests and plant pathogenic fungi so that they can be utilized as natural insecticides and antifungal agents [10,11]. In our continuing research to explore insecticidal and antifungal compounds from plant resources and to extend the knowledge towards the sesquiterpenoids molecules, the whole plants of *A. vestita* were collected and the extracts were carefully investigated. From the results of our studies on this species, we report herein the isolation, structural determination, and bioactivity of four new sesquiterpenoids, artemivestinolide D–G (**1**–**4**), along with three known metabolites, 1α-acetoxyeudesm-4-en-6β,11βH-12,6-olide (**5**) [6], dehydrocostus lactone (**6**) [12], and dihydroestafiatone (**7**) [13] (Figure 1). Herein, we reported the isolation and structural elucidation of the undescribed sesquiterpenoids by extensive spectroscopic techniques (Supplementary Materials) and chemical means. The CD exciton chirality method and calculated ECD spectra were used to determine the absolute configurations of the new compounds. The antifeedant activities against 3rd instar larvae of *Plutella xylostella* and antifungal activities for plant pathogenic fungi of the isolated compounds were evaluated.

## 2. Results

**Figure 2:**
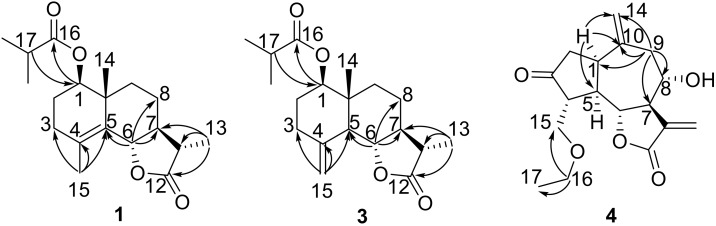
Key HMBC correlations of compounds **1**, **3**, and **4**.

**Figure 3:**
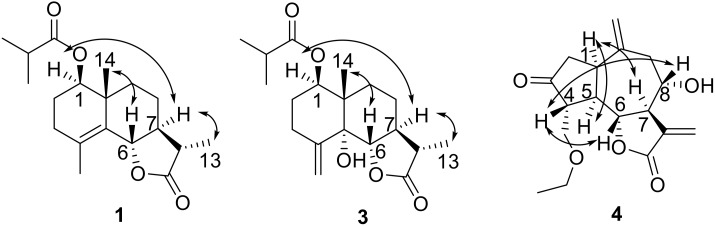
Key NOESY correlations of compounds **1**, **3**, and **4**.

**Figure 4:**
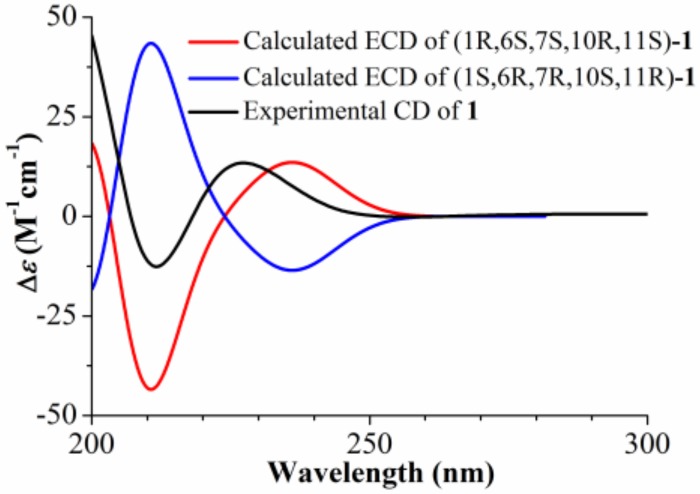
Calculated and experimental ECD spectra of compound **1**.

Compound **1** was obtained as a colorless gum and the molecular formula was established as C_19_H_28_O_4_ by HR-ESI-MS at *m*/*z* 343.1881 ([M + Na]^+^; calcd. 343.1880). The IR spectrum of **1** showed the characteristic absorption bands for carbonyl groups (1782 and 1729 cm^−1^) and double bond (1625 cm^−1^). The ^1^H NMR data of **1** (Table 1) exhibited the appearance of one secondary methyl at *δ*_H_ 1.23 (d, *J* = 7.2 Hz, H_3_-13), two tertiary methyl groups at *δ*_H_ 1.19 (s, H_3_-14) and 1.85 (s, H_3_-15), and two oxymethine protons at *δ*_H_ 4.75 (dd, *J* = 11.5, 2.8 Hz, H-1) and *δ*_H_ 4.55 (d, *J* = 11.2 Hz, H-6). Furthermore, methylene proton at *δ*_H_ 2.55 (m, H-17) and two secondary methyl groups at *δ*_H_ 1.17 (d, *J* = 7.0 Hz, H_3_-18) and 1.18 (d, *J* = 7.0 Hz, H_3_-19) revealed the presence of an isobutyrate group in the molecule. The ^13^C NMR spectrum (Table 1) indicated 19 carbon resonances, including two ester carbonyls at *δ*_C_ 178.7 (C-12) and 176.6 (C-16), two olefinic carbons at *δ*_C_ 126.4 (C-4) and 128.6 (C-5), and two oxygenated carbons at *δ*_C_ 78.9 (C-1) and 82.6 (C-6). The above NMR spectroscopic data and the degrees of unsaturation suggested that this compound was a sesquiterpene lactone containing three rings. Considering its biological source, it should be an eudesm-12,6-olide derivative [6]. In the HMBC spectrum (Figure 2), correlations were observed from H-1 to C-16 and from H-17 to C-1, indicating that an isobutyrate group was attached to C-1. The HMBC correlations of H_3_-15 to C-3, C-4, and C-5 allowed the assignment of the olefinic carbons at C-4 and C-5. The HMBC correlations of H-6 to C-5, C-7, and C-8 confirmed the 12,6-olide. The relative stereochemistry of **1** was established by the NOESY spectrum (Figure 3). The NOESY correlations between H_3_-14 with H-6, and between H-7 with H-1 and H_3_-13 indicated that H_3_-14 and H-6 were β-oriented, and H-1, H-7, and H_3_-13 were α-oriented (Figure 2). The absolute configuration at C-6 of **1** was determined to be S from the CD spectrum using empirical rules of π–π * transition of α,β-unsaturated γ-lactones [6,14], in which a negative Cotton effect was observed at 212 nm. Furthermore, the absolute configuration of **1** was assigned as (1R,6S,7S,10R,11S) based on the fact that the calculated ECD curve for (1R,6S,7S,10R,11S)-**1** agreed well with the experimental spectrum for **1** (Figure 4). Thus, compound **1** was elucidated as (1R,6S,7S,10R,11S)-1-isobutyryloxy-eudesma-4(5)-en-12,6-olide, named artemivestinolide D (**1**).

Compound **2** was obtained as a colorless gum and the molecular formula was established as C_19_H_26_O_4_ by HR-ESI-MS at *m*/*z*341.1721 ([M + Na]^+^; calcd. 341.1723). The NMR data of **2** (Table 1) closely resembled those of **1**. The only remarkable difference was a metharcylate group (*δ*_H_ 6.10, 5.57 and 1.96; *δ*_C_ 136.8, 125.3, and 18.3) at C-1 (*δ*_C_ 79.7). This assignment was corroborated by the HMBC correlations of H-1 (*δ*_H_ 4.80, t, *J* = 7.8 Hz) with C-16 (*δ*_C_ 168.9). The NOESY correlations between H_3_-14 (*δ*_H_ 1.20, s) with H-6 (*δ*_H_ 4.57, d, *J* = 11.2 Hz), and between H-7 (*δ*_H_ 1.68, m) with H-1 and H_3_-13 (*δ*_H_ 1.22, d, *J* = 7.2 Hz) indicated H_3_-14 and H-6 were β-oriented, and H-1, H-7, and H_3_-13 were α-oriented. The absolute configuration at C-6 of **2** was determined as S based on the negative Cotton effect at 211 nm compared with compound **1** in the CD experimental spectrum [6,14]. Thus, compound **2** was elucidated as (1R, 6S, 7S, 10R, 11S)-1-methacryloyloxy-eudesma-4(5)-en-12,6-olide, named artemivestinolide E (**2**).

**Figure 5:**
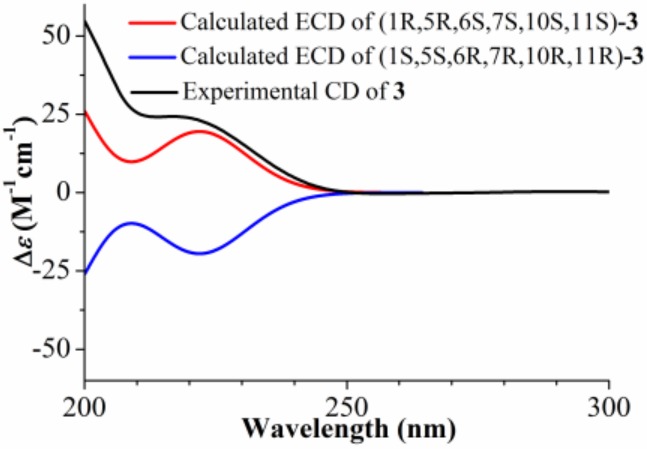
Calculated and experimental ECD spectra of compound **3**.

Compound **3** was obtained as a colorless gum and the molecular formula was established as C_19_H_28_O_5_ by HR-ESI-MS at *m*/*z* 354.2280 ([M + NH_4_]^+^; calcd. 354.2275). The overall appearance of ^1^H and ^13^C NMR spectra of **3** are highly similar to those of artemivestinolide C [6]. The most obvious distinction is an isobutyrate group (*δ*_H_ 2.51, 1.16, and 1.17; *δ*_C_ 176.3, 34.4, 19.0, and 19.1) at C-1 (*δ*_C_ 74.1). The cross peaks at H-1 (*δ*_H_ 5.34, dd, *J* = 11.0, 4.5 Hz) with C-16 in the HMBC spectrum confirmed this (Figure 2). The β-orientation was assigned to C-14 and α-orientation was assigned to 5-OH based on ^1^H NMR signals of H-1 and H_3_-14 and ^13^C NMR signals of C-5, C-10, and C-14 by comparing those with the literature data of eudesmane-type sesquiterpenoid [15]. The NOESY correlations between H_3_-14 (*δ*_H_ 1.00, s) with H-6 (*δ*_H_ 4.25, d, *J* = 9.8 Hz), and between H-7 (*δ*_H_ 2.39, m) with H-1 and H_3_-13 (*δ*_H_ 1.23, d, *J* = 7.2 Hz) indicated that H_3_-14 and H-6 were β-oriented, and H-1, H-7, and H_3_-13 were α-oriented (Figure 3). The absolute configuration at C-5 of **3** was determined as R based on the positive Cotton effect at about 200 nm in the CD experimental spectrum [6]. Furthermore, the absolute configuration of **3** was assigned as (1R,5R,6S,7S,10S,11S) based on the calculated ECD curve for (1R,5R,6S,7S,10S,11S)-**3** agreed well with the experimental spectrum for **3** (Figure 5). Thus, compound **3** was elucidated as (1R,5R,6S,7S,10S,11S)-1-isobutyryloxy-eudesma-4(15)-en-12,6-olide, named artemivestinolide F (**3**).

**Figure 6:**
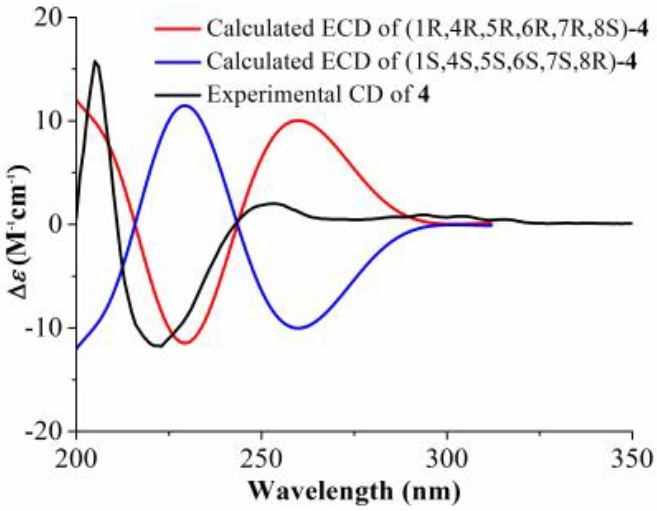
Calculated and experimental ECD spectra of compound **4.**

Compound **4**, obtained as a pale yellow oil, was found to have the molecular formula C_17_H_22_O_5_, as determined through HR-ESI-MS ([M + H]^+^ peak at *m*/*z* 307.1540, calc for C_17_H_23_O_5_, 307.1546). The IR spectrum indicated a hydroxyl group (3431 cm^−1^) and a carbonyl group (1738 cm^−1^). The ^1^H NMR spectrum of **4** (Table 1) showed signals of two oxygenated methane protons at *δ*_H_ 4.02 (t, *J* = 8.6 Hz, H-6) and 3.94 (m, H-8), two oxygenated methylene protons at *δ*_H_ 3.91 (dd, *J* = 9.1, 2.7 Hz, H-15a), 3.70 (dd, *J* = 9.1, 2.7 Hz, H-15b), and 3.47 (m, H_2_-16), and two terminal double bonds at *δ*_H_ 6.39 (s, H-13a), 6.31 (s, H-13b), 5.08 (s, H-14a), and 4.87 (s, H-14b). The ^13^C NMR spectrum (Table 1) indicated 17 C-atom signals which were classified by a DEPT experiment into one ketone carbonyl at *δ*_C_ 216.8 (C-3), one unsaturated ester carbonyl at *δ*_C_ 169.6 (C-12), two terminal olefinic carbons at *δ*_C_ 125.2 (C-13) and 115.9 (C-14), and four oxygenated carbons at *δ*_C_ 81.8 (C-6), 73.1 (C-8), 68.9 (C-15), and 66.7 (C-16). The above information suggested that compound **4** was a guaianolide and similar to grosheimin [16] except for a ethoxymethyl group in **4**. In the HMBC spectrum (Figure 2), correlations were observed from H_2_-16 to C-15 and C-17; from H-1 to C-5, C-10, and C-14; and from H_2_-9 to C-1, C-7, C-8, C-10, and C-14, indicating that the ethoxymethyl group was attached to C-15. The relative configuration of **4** was established by the NOESY experiment, by which the correlations from H-4 to H-6 and H-8, and from H-1 to H-5 and H-7, indicated that H-4, H-6, and H-8 were β-oriented, and H-1, H-5, and H-7 were α-oriented (Figure 3). The absolute configuration of **4** was assigned as (1R,4R,5R,6R,7R,8S) based on the fact that the calculated ECD curve for (1R,4R,5R,6R,7R,8S)-**4** agreed well with the experimental spectrum for **4** (Figure 6). The chemical structure of compound **4** was elucidated as (1R,4R,5R,6R,7R,8S)-15-ethoxymethyl-2-oxo-8-hydroxy-guaia-10(14),11(13)-dien-12,6-olide, named artemivestinolide G (**4**).

Seven sesquiterpenoids were isolated from *A. vestita*, including three new eudesmanolides (**1**–**3**) and one new guaianolide (**4**). The result was in agreement with the report suggesting that the sesquiterpenoids were the main secondary metabolites from *A. vestita* [6,7]. The quality of the sesquiterpenoids we obtained was insufficient and more experiments should be done to optimize the extraction and separation of these compounds.

The isolated sesquiterpenoids were preliminarily investigated for their antifeedant activities against third-instar larvae of *P. xylostella* and antifungal activities against three pathogenic fungi (*Pyricularia oryzae*, *Botrytis cinerea*, and *Fusarium oxysporum*). As shown in Table 2, all compounds were found to have potential deterrence against the *P. xylostella*, with EC_50_ values ranging from 25.3–42.1 μg/cm^2^. Furthermore, some sesquiterpenoids showed antifungal activities against pathogenic fungi. For *P. oryzae*, compounds **1** and **6** displayed antifungal activities (MIC of 128 mg/L). For *B. cinerea*, compounds **1**, **2**, **4**, and **7** showed antifungal effects (MIC of 256 mg/L). For *F. oxysporum*, compounds **3** and **6** displayed antifungal activities (MIC of 256 mg/L). These results may reveal a way to search for natural product inspired synthetic insecticides and antifungal agents.

## 3. Materials and Methods

### 3.1. General Experimental Material

Optical rotation data were measured on an Anton Paar MCP 200 Analytical Automatic Polarimeter (Anton Paar GmbH, Graz, Austria). IR data were recorded using a Nicolet Magna-IR 750 spectrophotometer (Thermo Scientific, San Jose, CA, USA). NMR spectra were recorded on AV-600 spectrometer (Bruker, Fällanden, Switzerland). ^1^H-^1^H COSY, HSQC, HMBC, and NOESY spectra were measured with the pulse sequence cosygpqf, hsqcetgpsi2, hmbcgpndqf, and noesygpph, respectively (standard sequence, Bruker TOPSPIN 3.1 software) (Bruker, Fällanden, Switzerland). The mixing time (D8) of NOESY spectra was 400 ms. HR-ESI-MS were measured by an Agilent 6520 Q-TOF LC-MS mass spectrometer (Agilent Technologies Co., Ltd., Palo Alto, CA, USA). Analytical and preparative TLC were run on silica gel plates (GF_254_, Qingdao Haiyang Chemical Co., Ltd., Qingdao, China). Spots were visualized by spraying with 10% H_2_SO_4_, followed by heating. Column chromatography (CC) was performed on silica gel (200-300 mesh, Qingdao Haiyang Chemical Co., Ltd., Qingdao, China) and Sephadex LH-20 (Pharmacia Fine Chemical Co., Ltd., Uppsala, Sweden). Acarbose was purchased from Sigma-Aldrich (St. Louis, MO, USA).

### 3.2. Plant Material

The *A. vestita* was collected in Alxa left Banner, Inner Mongolia Autonomous Region, People’s Republic of China, in August 2017, and identified by one of the authors (H.-B.W.). A voucher specimen (NO. 20170839) was deposited in the herbarium of the College of Life and Environmental Sciences, Minzu University of China, Beijing, People’s Republic of China.

### 3.3. Extraction and Isolation

The chopped and dried *A. vestita* (5.0 kg) were pulverized and extracted three times with MeOH (each for seven days) at room temperature. After filtration, the filtrate was concentrated under reduced pressure to yield a residue (225.0 g ) and then was suspended in water and partitioned successively with petroleum ether, CHCl_3_, EtOAc, and *n*-butanol, to afford five fractions. The CHCl_3_ fraction (45.5 g) was subjected to silica gel CC (1.0 kg) eluting with PE−CHCl_3_ (15:1−0:1, gradient system). On the basis of TLC analysis, five fractions A−E were obtained. Fraction A (4.2 g) was eluted with PE−EtOAc (12:1) on CC, further purified by Sephadex LH-20 (CHCl_3_−MeOH, 1:1) to afford **1** (6.1 mg)**, 2** (4.1 mg), and **5** (3.9 mg). Fraction B (1.6 g) was subjected to CC eluting with PE−EtOAc (8:1) to afford **3** (3.3 mg)**,** and **6** (1.9 mg). Fraction C (1.9 g) was subjected to CC eluting with PE−CHCl_3_ (3:1) to afford **4** (1.6 mg) and **7** (2.2 mg) [17].

### 3.4. Spectroscopic Data

*Artemivestinolide D* (**1**): Colorless gum; ${\left[\alpha \right]}_{D}^{20}\;$ +24.5 (*c* = 0.12, MeOH); UV (CH_3_CN) *λ*_max_ (log *ε*) 200 (2.42) nm; ECD (CH_3_CN) *λ*_max_ (Δ*ε*) 212 (−5.1), 227 (+5.37) nm; IR (KBr) *ν*_max_ 1782, 1729, 1625, 1157 cm^−1^; ^1^H and ^13^C NMR data (see Table 1); HR−ESI−MS *m*/*z* 343.1881 (calcd for C_19_H_28_NaO_4_, 343.1880).

*Artemivestinolide E* (**2**): Colorless gum; ${\left[\alpha \right]}_{D}^{20}$ +27.6 (*c* = 0.16, MeOH); UV (CH_3_CN) *λ*_max_ (log *ε*) 231 (3.39) nm; ECD (CH_3_CN) *λ*_max_ (Δ*ε*) 211 (−34.4), 233 (+2.94) nm; IR (KBr) *ν*_max_ 1781, 1716, 1634, 1166 cm^−1^; ^1^H and ^13^C NMR data (see Table 1); HR−ESI−MS *m*/*z* 341.1721 (calcd for C_19_H_26_NaO_4_, 341.1723).

*Artemivestinolide F* (**3**): Colorless gum; ${\left[\alpha \right]}_{D}^{20}$ +17.3 (*c* = 0.13, MeOH); UV (CH_3_CN) *λ*_max_ (log *ε*) 200 (1.22) nm; ECD (CH_3_CN) *λ*_max_ (Δ*ε*) 200 (+45.7) nm; IR (KBr) *ν*_max_ 1773, 1726, 1613, 1157 cm^−1^; ^1^H and ^13^C NMR data (see Table 1); HR−ESI−MS *m*/*z* 354.2280 (calcd for C_19_H_32_NO_5_, 354.2275).

*Artemivestinolide G* (**4**): Pale yellow oil; ${\left[\alpha \right]}_{D}^{20}$ +6.7 (*c* = 0.07, MeOH); UV (CH_3_CN) *λ*_max_ (log *ε*) 210 (0.35) nm; ECD (CH_3_CN) *λ*_max_ (Δ*ε*) 205 (+27.6), 223 (−20.6), 253 (+3.5) nm; IR (KBr) *ν*_max_ 3431, 1738, 1634, 1385, 1120 cm^−1^; ^1^H and ^13^C NMR data (see Table 1); HR−ESI−MS *m*/*z* 307.1540 (calcd for C_17_H_23_O_5_, 307.1546).

### 3.5. Antifeedant Bioassay

The antifeeding effect was estimated through a no-choice assay using leaf disc method [11]. Leaf discs (1 cm × 1 cm) of cabbage were cut and immersed in acetone solution with compounds for 5 s, and air dried at room temperature. Five treated leaf discs and 10 larvae were placed together in one 90 mm diameter Petri dish. Residual leaf area of the control and the treatment were measured separately by graph paper at 24 h post treatment and the percentage antifeedant index was then determined using the following equation: antifeedant index (%) = [(CK−T)/CK] × 100, where CK refers to the leaf area consumed in the control disc while T indicates the leaf area consumed in the treatment.

### 3.6. Antifungal Bioassay

The test phytopathogenic fungi used in this study were *P. oryzae*, *B. cinerea*, and *F. oxysporum*. The microdilution method, with 96-well microliter plates using a potato dextrose (PD) medium, was used to evaluate antifungal activity of the compounds [18]. After incubation for 48 h at 28 ± 0.5 °C, minimum inhibitory concentration (MIC) was taken as the lowest concentration of the test compounds in 96-well plate, in which no microbial growth could be observed.

## 4. Conclusions

In this study, seven sesquiterpenoids, including four new compounds, were isolated from *A. vestita*, and their structures were primarily elucidated on the basis of NMR and MS studies. The absolute configuration of the new compounds was determined by CD exciton chirality and calculated ECD methods. The isolated compounds were tested for antifeedant activities against third-instar larvae of *P. xylostella* and antifungal activities against three pathogenic fungi, with all compounds showing moderate antifeedant activities and some compounds exhibiting antifungal activities.

## Figures and Tables

**Table 1:** ^1^H NMR (600 MHz) and ^13^C NMR (150 MHz) spectroscopic data for compounds **1**–**4** in CDCl_3_.

Position	1		2		3		4	
	*δ*_H_ (*J* in Hz)	*δ*_C_	*δ*_H_ (*J* in Hz)	*δ*_C_	*δ*_H_ (*J* in Hz)	*δ*_C_	*δ*_H_ (*J* in Hz)	*δ*_C_
1	4.75, dd (11.5, 2.8)	78.9	4.80, t (7.8)	79.7	5.34, dd (11.0, 4.5)	74.1	3.23, dt (8.8, 4.0)	40.9
2a	1.76, m	23.8	1.82, m	23.7	1.90, m	26.8	2.56, dd (18.2, 8.8)	44.7
2b	1.71, overlapped		1.80, m		1.61, m		2.46, dd (18.2, 4.0)	
3a	2.27, overlapped	32.8	2.27, overlapped	32.9	2.71, m	29.3		216.8
3b	2.01, m		2.03, m		2.16, m			
4		126.4		126.5		144.6	2.38, m	52.7
5		128.6		128.5		77.1	2.90, dd (17.6, 8.3)	45.9
6	4.55, d (11.2)	82.6	4.57, d (11.2)	82.6	4.25, d (9.8)	81.4	4.02, t (8.3)	81.8
7	1.70, overlapped	52.8	1.68, m	52.8	2.39, overlapped	45.3	3.08, m	49.7
8a	1.90, m	24.3	1.91, m	24.3	1.82, m	22.7	3.94, m	73.1
8b	1.49, m		1.51, m		1.47, overlapped			
9a	1.73, overlapped	38.0	1.76, overlapped	38.1	1.72, overlapped	29.8	2.86, dd (12.8, 5.6)	47.1
9b	1.28, m		1.28, m		1.46, overlapped		2.34, dd (12.8, 8.3)	
10		41.0		41.1		43.9		143.4
11	2.28, overlapped	41.0	2.27, overlapped	41.1	2.31, overlapped	41.2		136.9
12		178.7		178.7		179.1		169.6
13a	1.23, d (7.2)	12.4	1.22, d (7.2)	12.4	1.23, d (7.2)	12.4	6.39, s	125.2
13b							6.31, s	
14a	1.19, s	19.8	1.20, s	19.8	1.00, s	14.6	5.08, s	115.9
14b							4.87, s	
15a	1.85, s	19.8	1.82, s	19.8	5.55, s	112.7	3.91, dd (9.1, 2.7)	68.9
15b					5.00, s		3.70, dd (9.1, 2.7)	
16		176.6		168.9		176.3	3.47, m	66.7
17	2.55, m	34.5		136.8	2.51, m	34.4	1.16, t (7.0)	14.9
18	1.17, d (7.0)	18.9	6.10, s; 5.57, s	125.3	1.16, s	19.0		
19	1.18, d (7.0)	19.1	1.96, s	18.3	1.17, s	19.1		

**Table 2:** Antifeedant activity of compounds **1**–**7** against *P. xylostella.*

Compounds	EC_50_ (μg/cm^2^)	Compounds	EC_50_ (μg/cm^2^)
**1**	32.4	**5**	33.0
**2**	37.9	**6**	31.9
**3**	42.1	**7**	25.3
**4**	27.6	Neem oil	4.89

## Supplementary Materials

Supplementary material relating to this article can be accessed online.
