# Supplementary material for: Sesquiterpenoids from Artemisia vestita and Their Antifeedant and Antifungal Activities
Source: Molecules. 2019 Oct 11;24(20):3671. doi: 10.3390/molecules24203671 (PMC6832759; doi:10.3390/molecules24203671)
Supplement: Supplementary file 1 [file molecules-24-03671-s001.pdf]

Supporting Information

-List of Contents

No.	**Contents**
Figure S1.	^{1}H NMR (600 MHz, $CDCl_3$) spectrum of compound **1**
Figure S2.	^{13}C NMR (150 MHz, $CDCl_3$) spectrum of compound **1**
Figure S3.	^{1}H–^{1}H COSY spectrum ($CDCl_3$) of compound **1**
Figure S4.	HSQC spectrum ($CDCl_3$) of compound **1**
Figure S5.	HMBC spectrum ($CDCl_3$) of compound **1**
Figure S6.	NOESY spectrum ($CDCl_3$) of compound **1**
Figure S7.	HRESIMS spectrum of compound **1**
Figure S8.	^{1}H NMR (600 MHz, $CDCl_3$) spectrum of compound **2**
Figure S9.	^{13}C NMR (150 MHz, $CDCl_3$) spectrum of compound **2**
Figure S10	^{1}H–^{1}H COSY spectrum ($CDCl_3$) of compound **2**
Figure S11	HSQC spectrum ($CDCl_3$) of compound **2**
Figure S12	HMBC spectrum ($CDCl_3$) of compound **2**
Figure S13	NOESY spectrum ($CDCl_3$) of compound **2**
Figure S14	HRESIMS spectrum of compound **2**
Figure S15.	^{1}H NMR (600 MHz, $CDCl_3$) spectrum of compound **3**
Figure S16.	^{13}C NMR (150 MHz, $CDCl_3$) spectrum of compound **3**
Figure S17.	^{1}H–^{1}H COSY spectrum ($CDCl_3$) of compound **3**
Figure S18.	HSQC spectrum ($CDCl_3$) of compound **3**
Figure S19.	HMBC spectrum ($CDCl_3$) of compound **3**
Figure S20	NOESY spectrum ($CDCl_3$) of compound **3**
Figure S21	HRESIMS spectrum of compound **3**
Figure S22.	^{1}H NMR (600 MHz, $CDCl_3$) spectrum of compound **4**
Figure S23.	^{13}C NMR (150 MHz, $CDCl_3$) spectrum of compound **4**
Figure S24.	^{1}H–^{1}H COSY spectrum ($CDCl_3$) of compound **4**
Figure S25	HSQC spectrum $CDCl_3$) of compound **4**
Figure S26	HMBC spectrum ($CDCl_3$) of compound **4**
Figure S27	NOESY spectrum ($CDCl_3$) of compound **4**
Figure S28	HRESIMS spectrum of compound **4**
	The ECD calculation details of compounds **1**, **3** and **4**
Figure S29.	B3LYP/6-31G (d) optimized lowest energy conformers for (1R,6S,7S,10R,11S)-**1**
Figure S30.	B3LYP/6-31G (d) optimized lowest energy conformers for (1R,5R,6S,7S,10S,11S)-**3**
Figure S31	B3LYP/6-31G (d) optimized lowest energy conformers for (1R,4R,5R,6R,7R,8S)-**4**

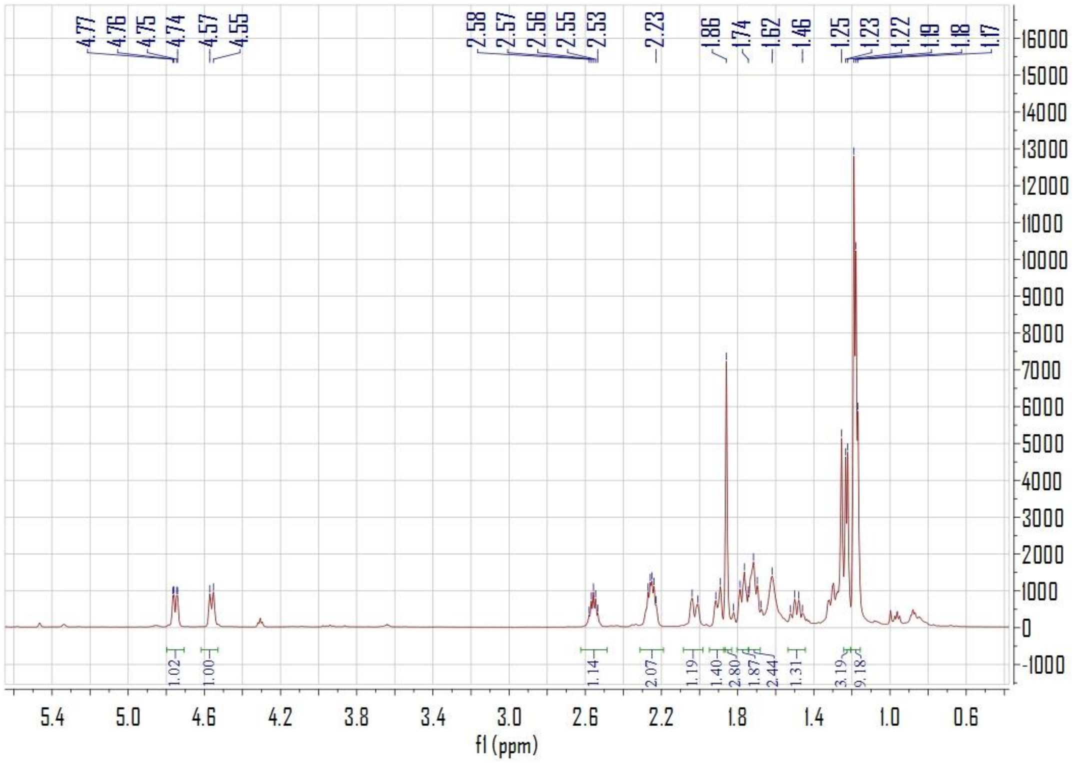

Figure S1. 1H NMR spectrum of compound 1

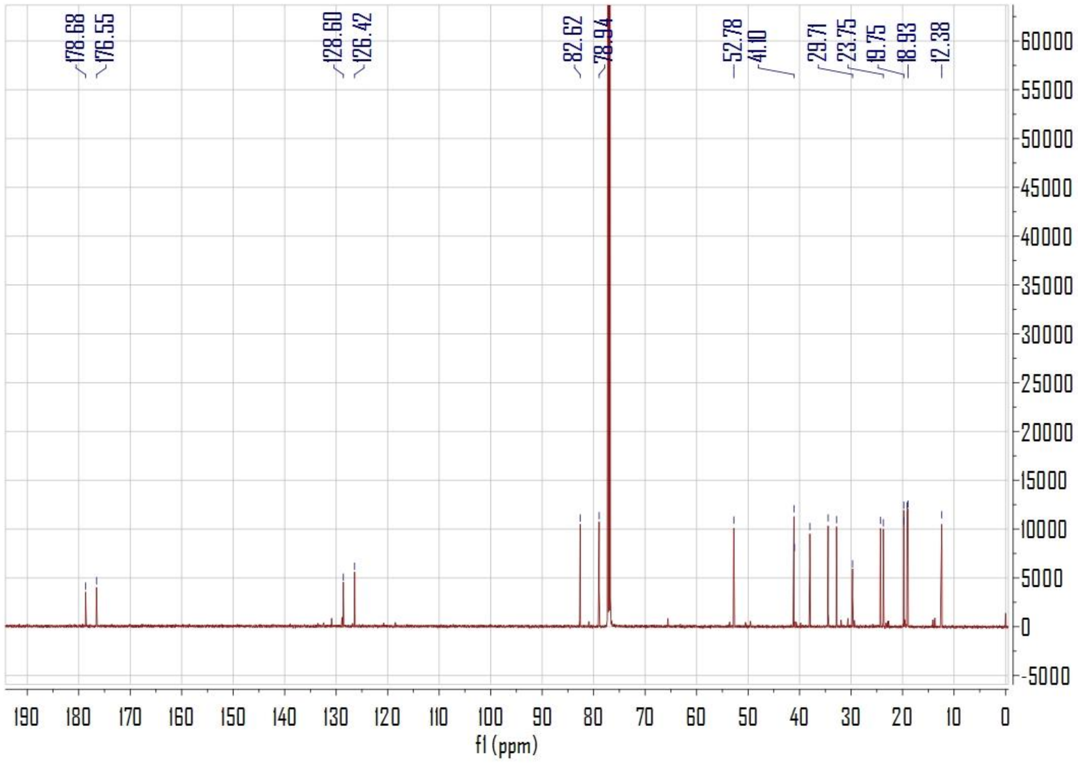

Figure S2. 13C NMR spectrum of compound 1

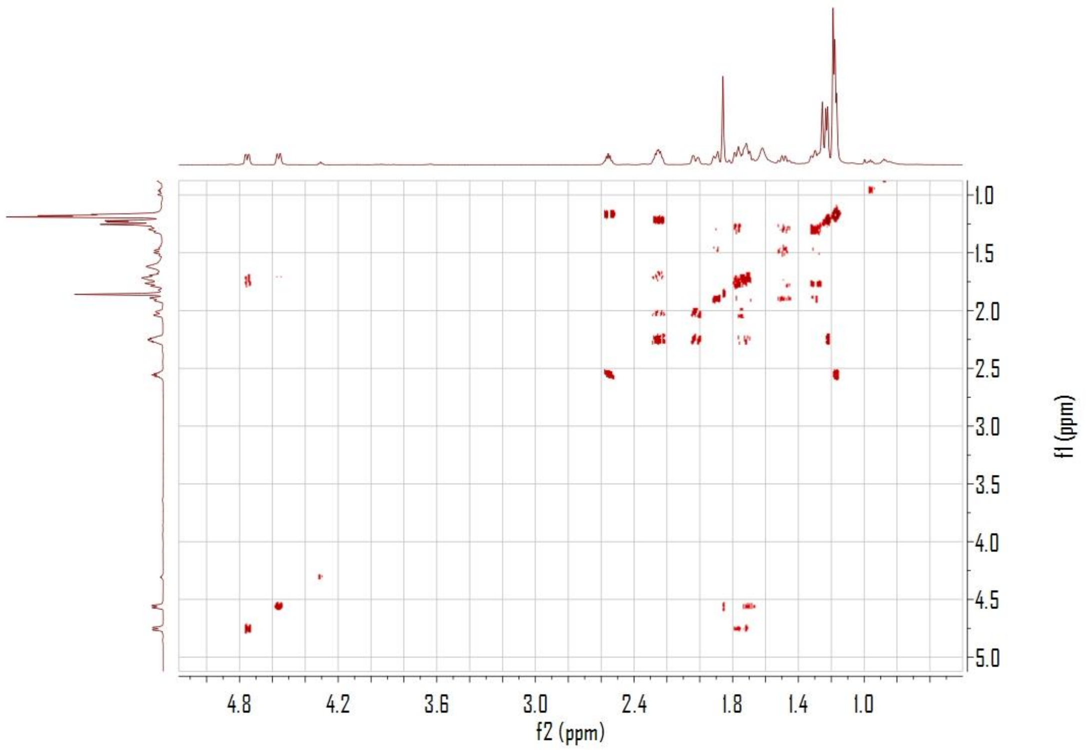

Figure S3. 1H–1H COSY spectrum of compound 1

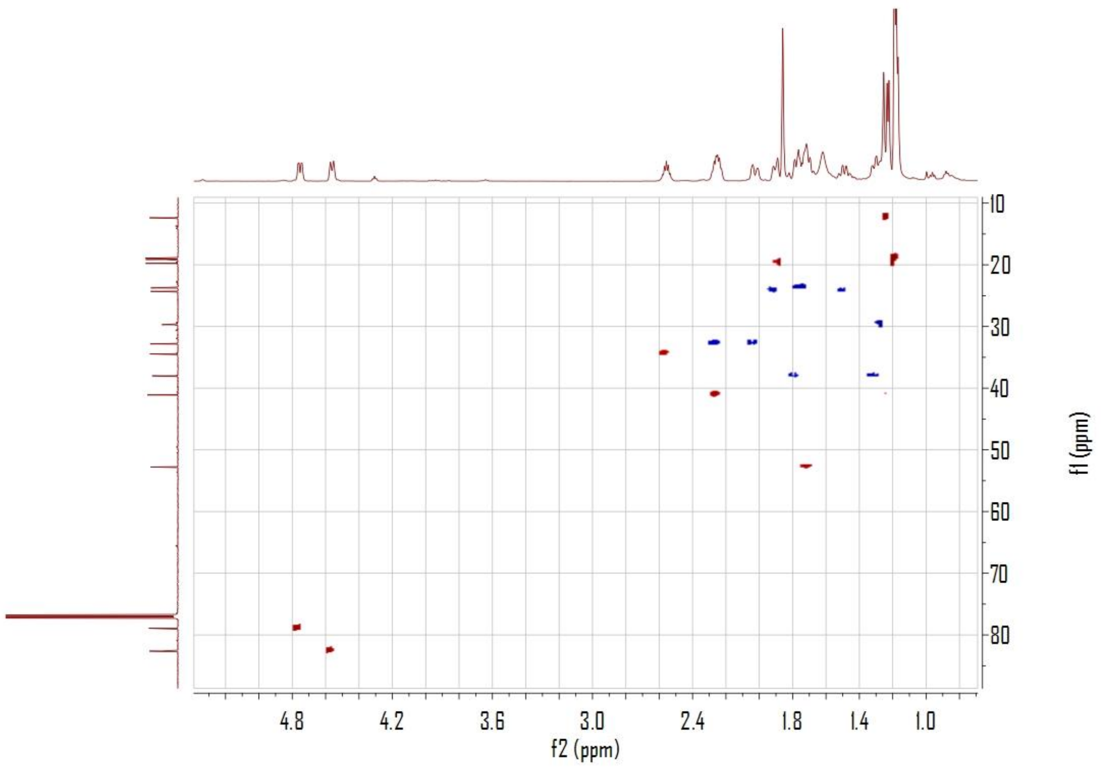

Figure S4. HSQC spectrum of compound 1

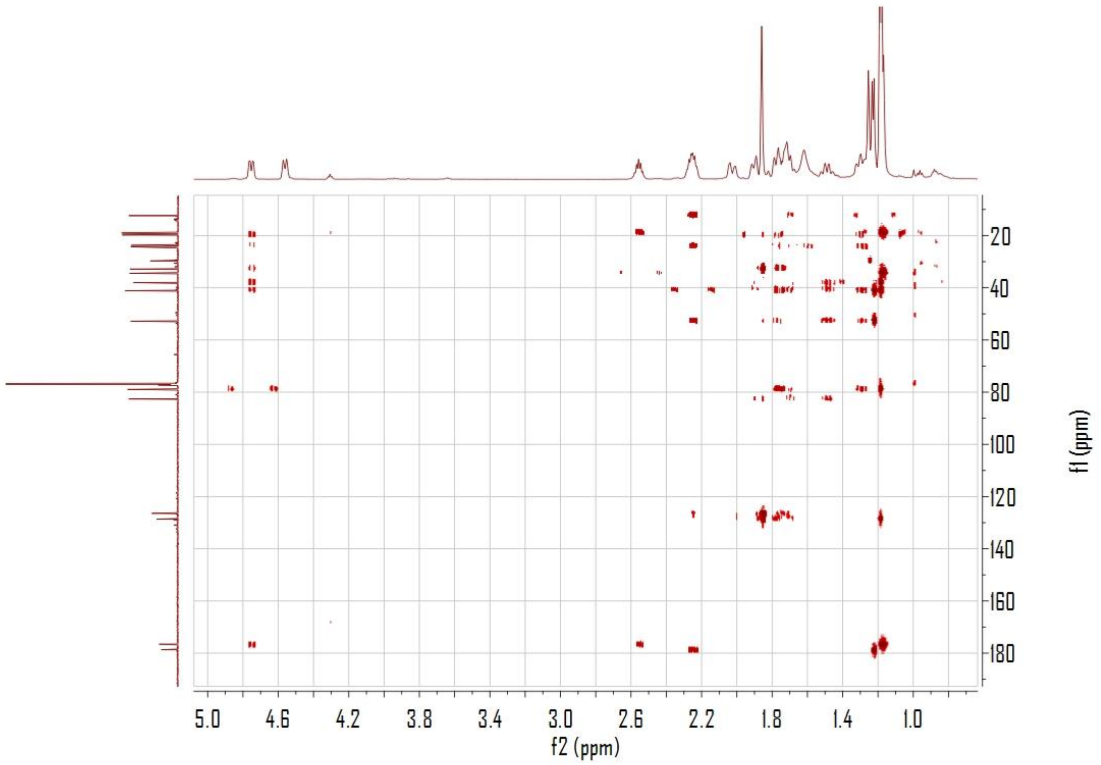

Figure S5. HMBC spectrum of compound 1

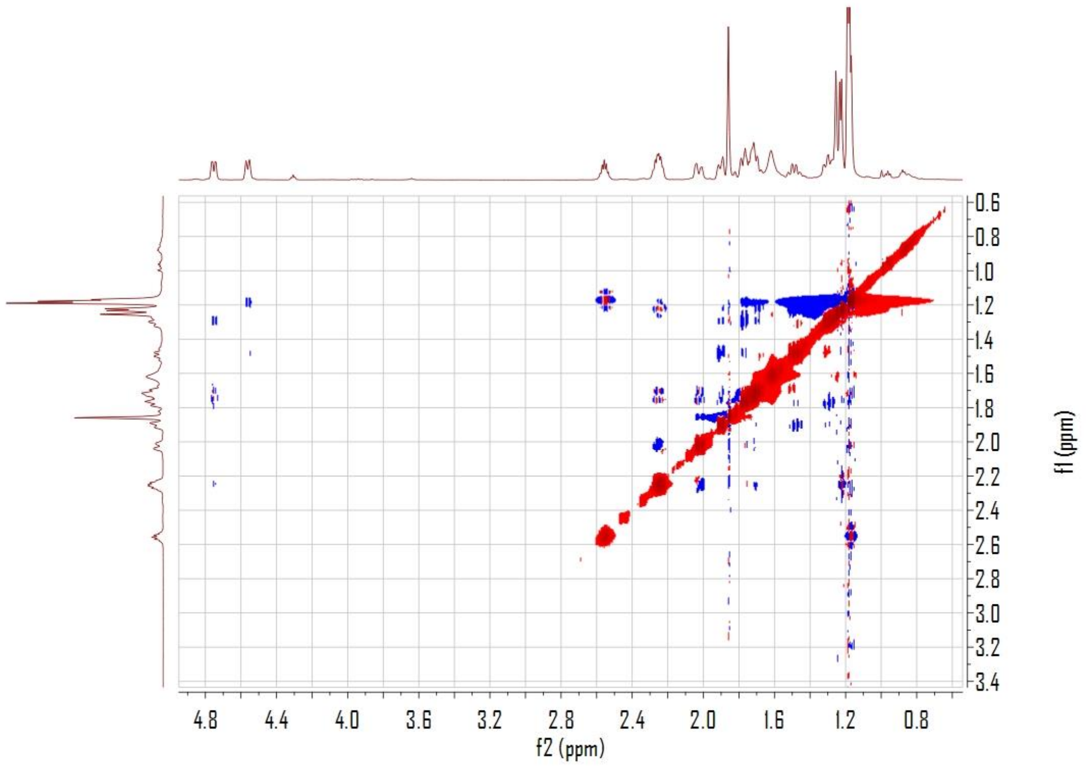

Figure S6. NOESY spectrum (CDCl3) of compound 1

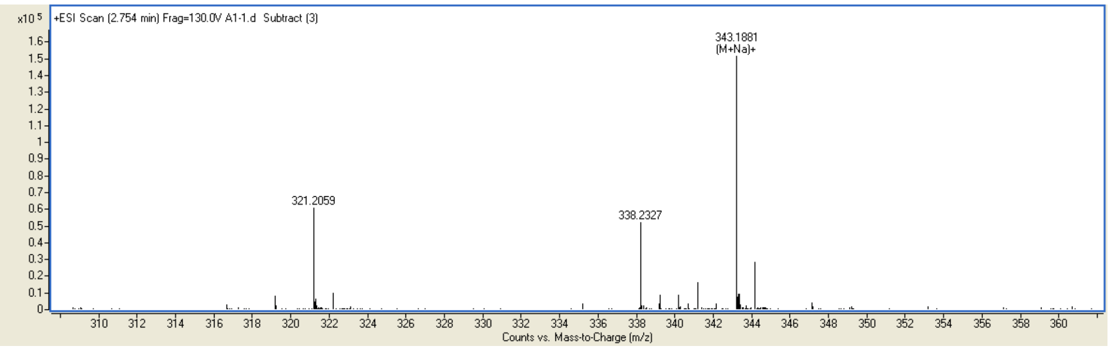

Figure S7. HRESIMS spectrum of compound 1

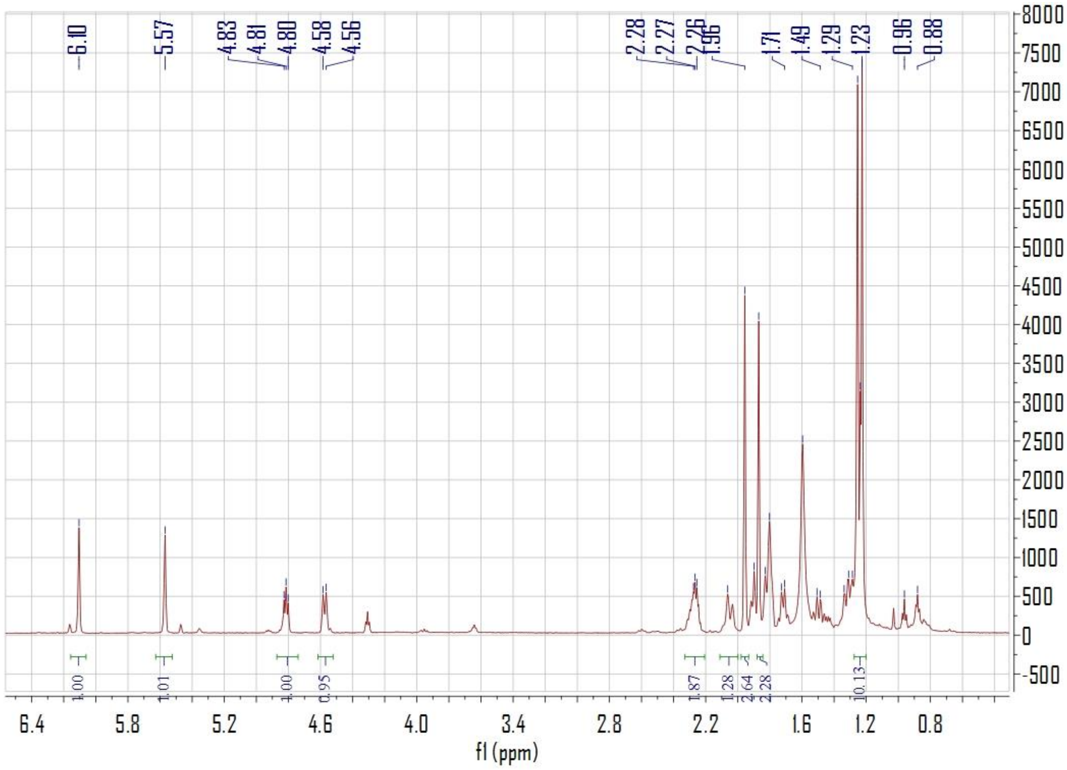

Figure S8. 1H NMR (600 MHz, CDCl3) spectrum of compound 2

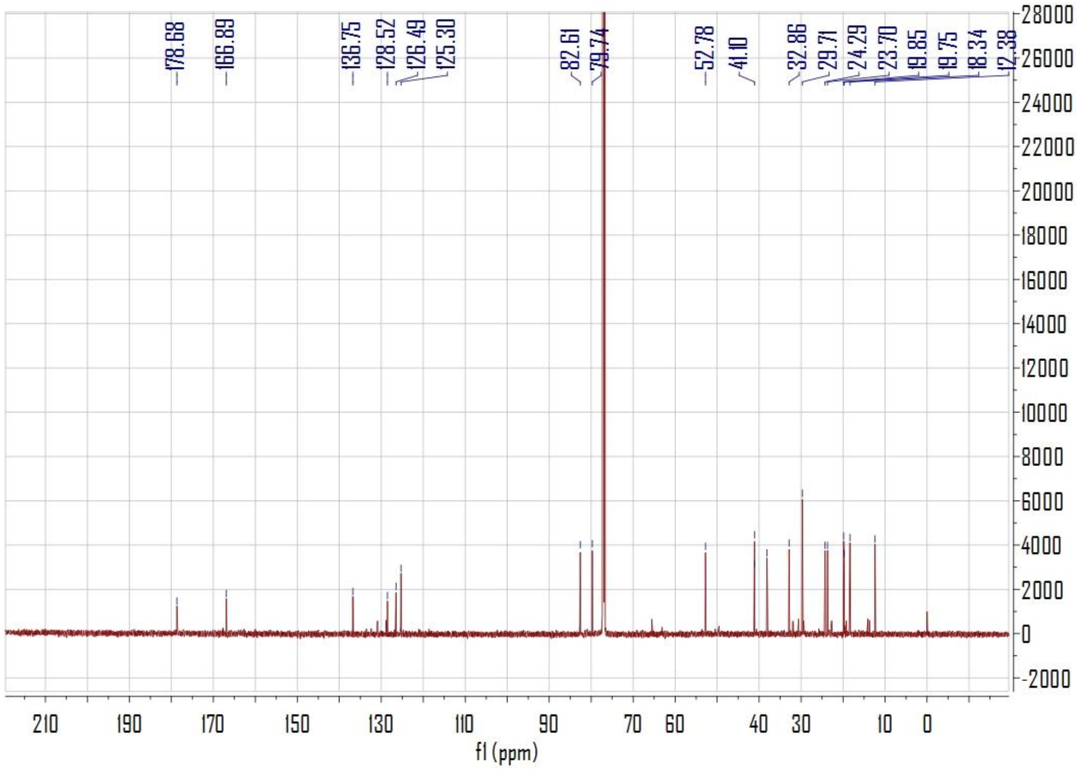

Figure S9. 13C NMR (150 MHz, CDCl3) spectrum of compound 2

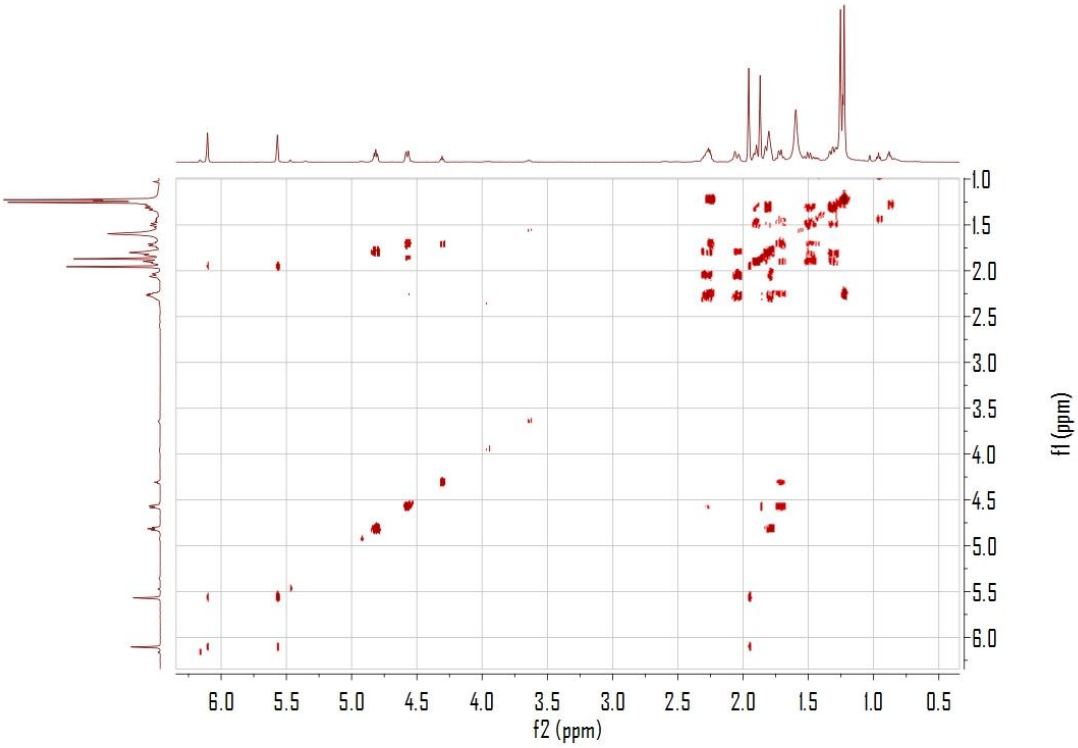

Figure S10 1H–1H COSY spectrum (CDCl3) of compound 2

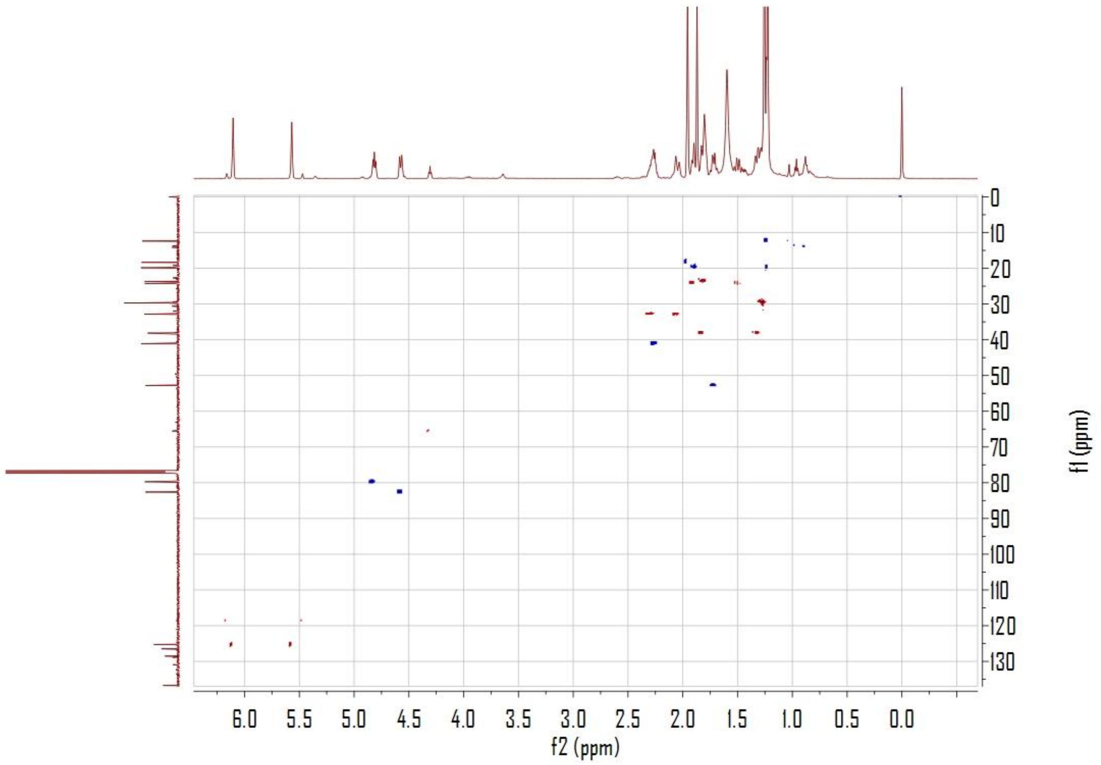

Figure S11 HSQC spectrum (CDCl3) of compound 2

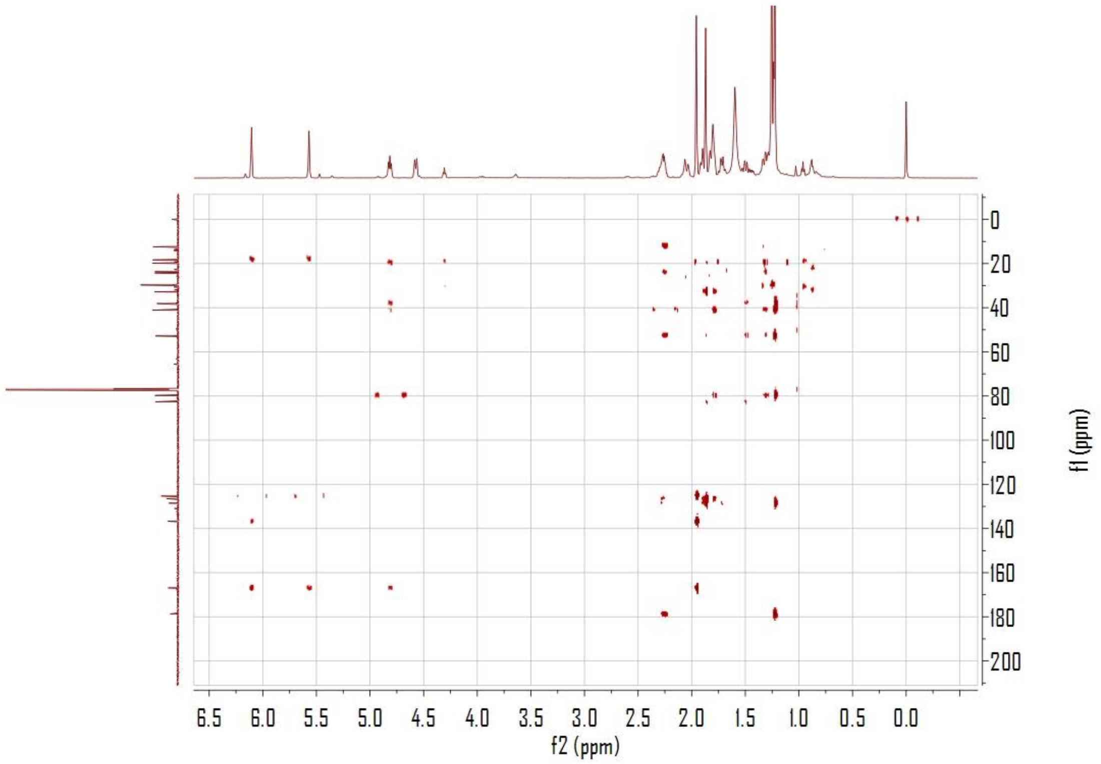

Figure S12 HMBC spectrum (CDCl3) of compound 2

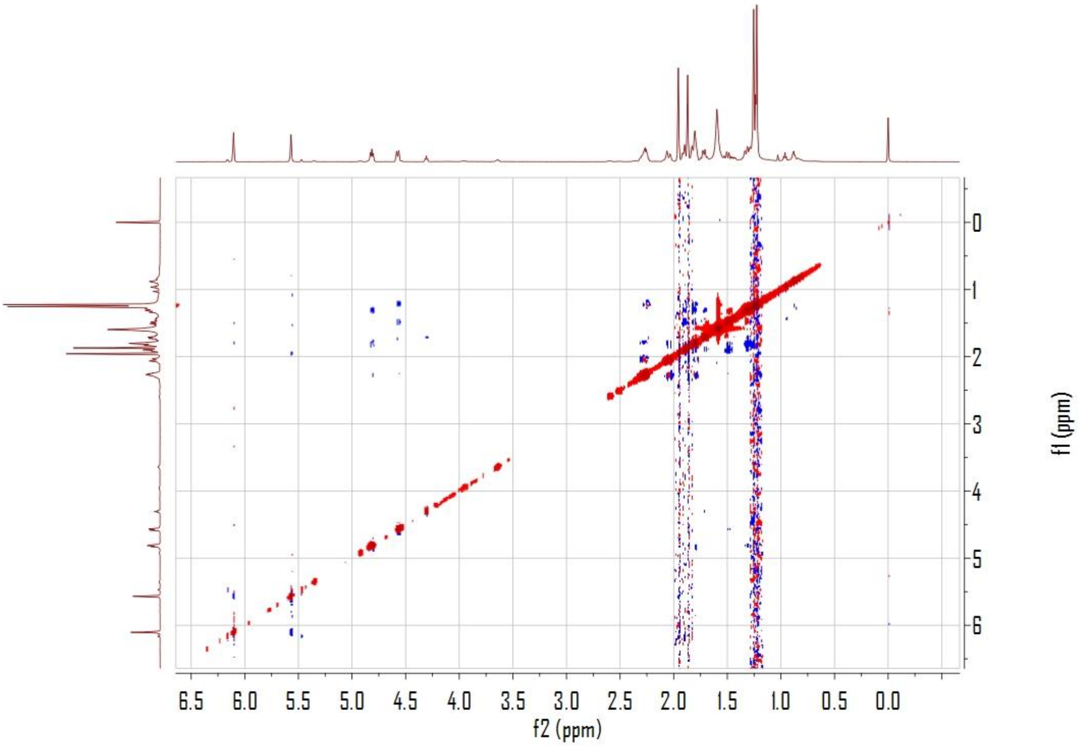

Figure S13 NOESY spectrum (CDCl3) of compound 2

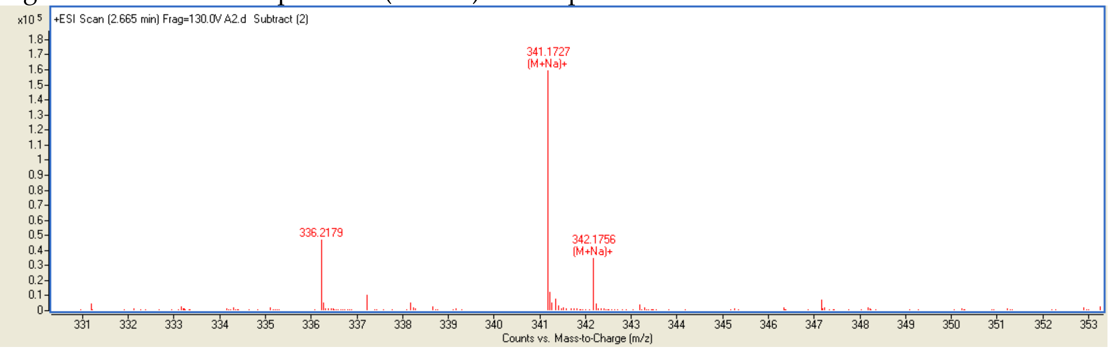

Figure S14 HRESIMS spectrum of compound 2

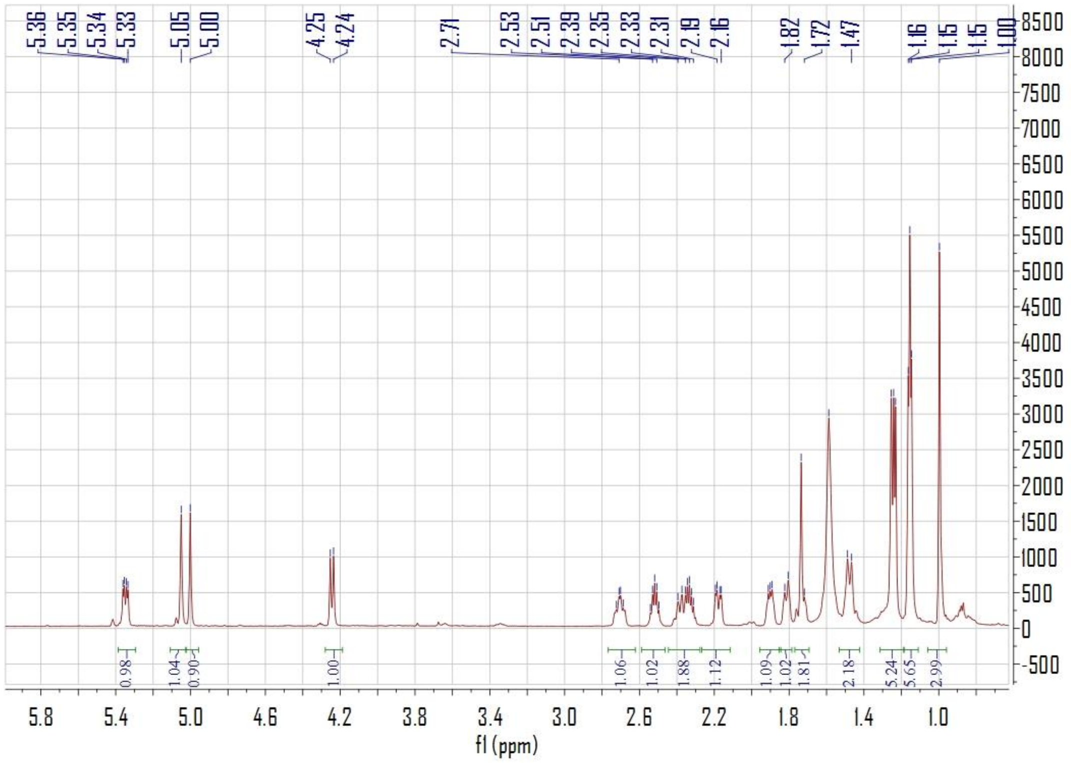

Figure S15. 1H NMR (600 MHz, CDCl3) spectrum of compound 3

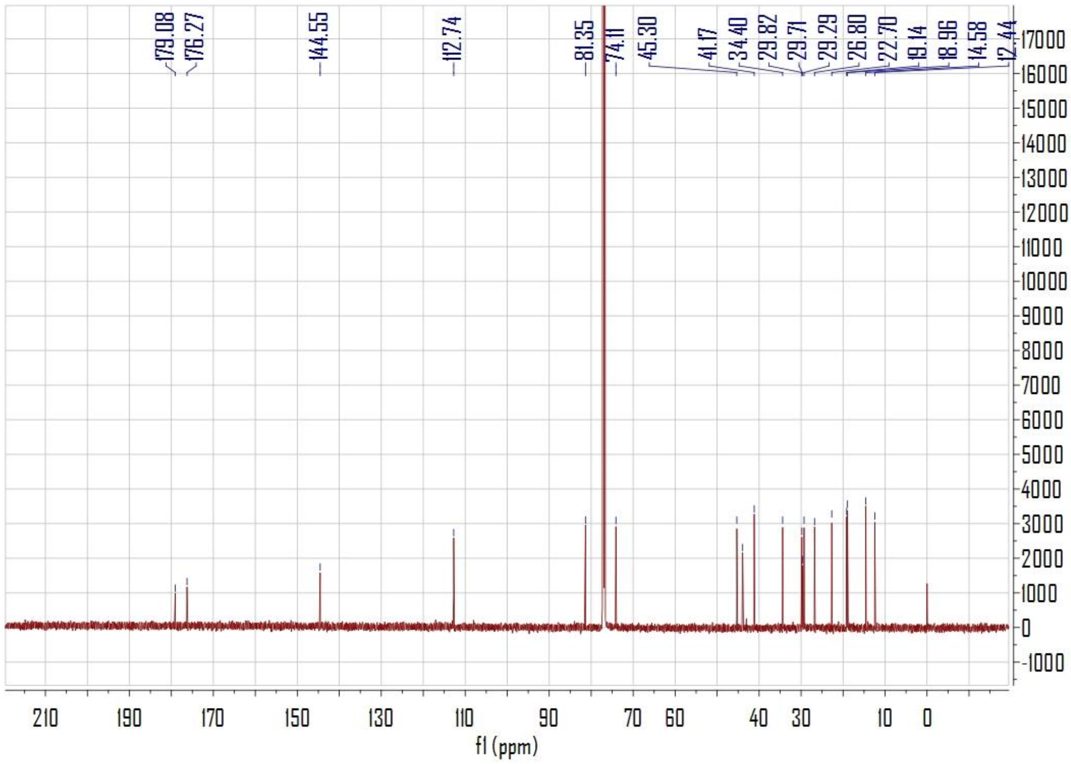

Figure S16. 13C NMR (150 MHz, CDCl3) spectrum of compound 3

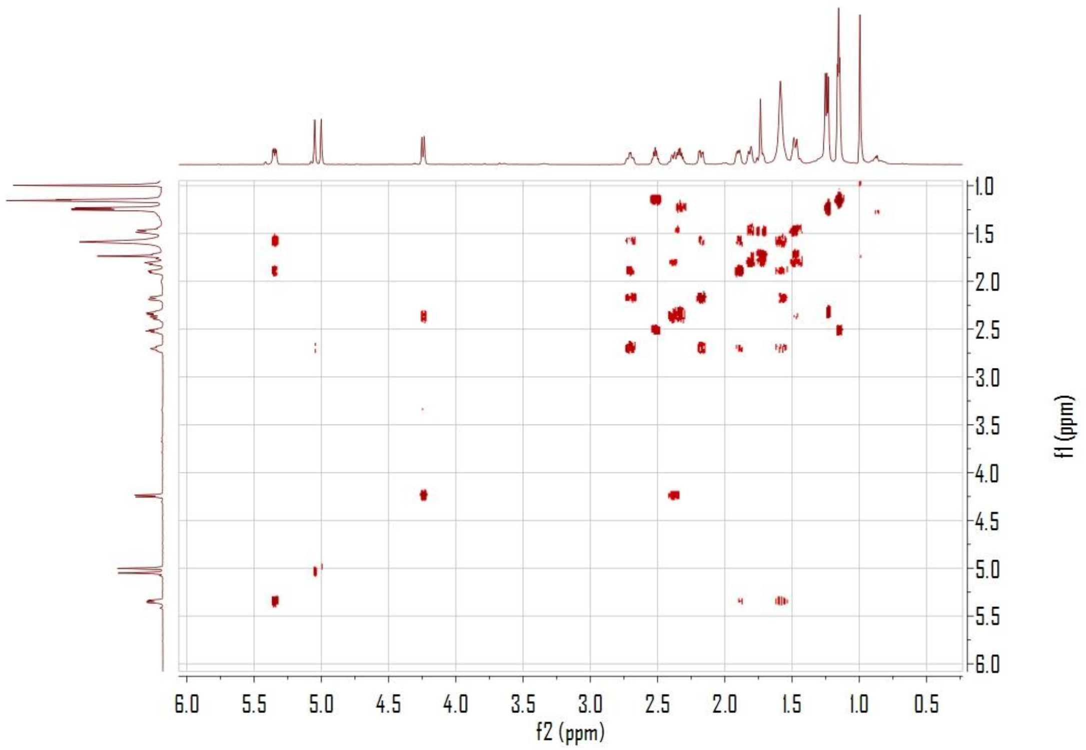

Figure S17. 1H–1H COSY spectrum (CDCl3) of compound 3

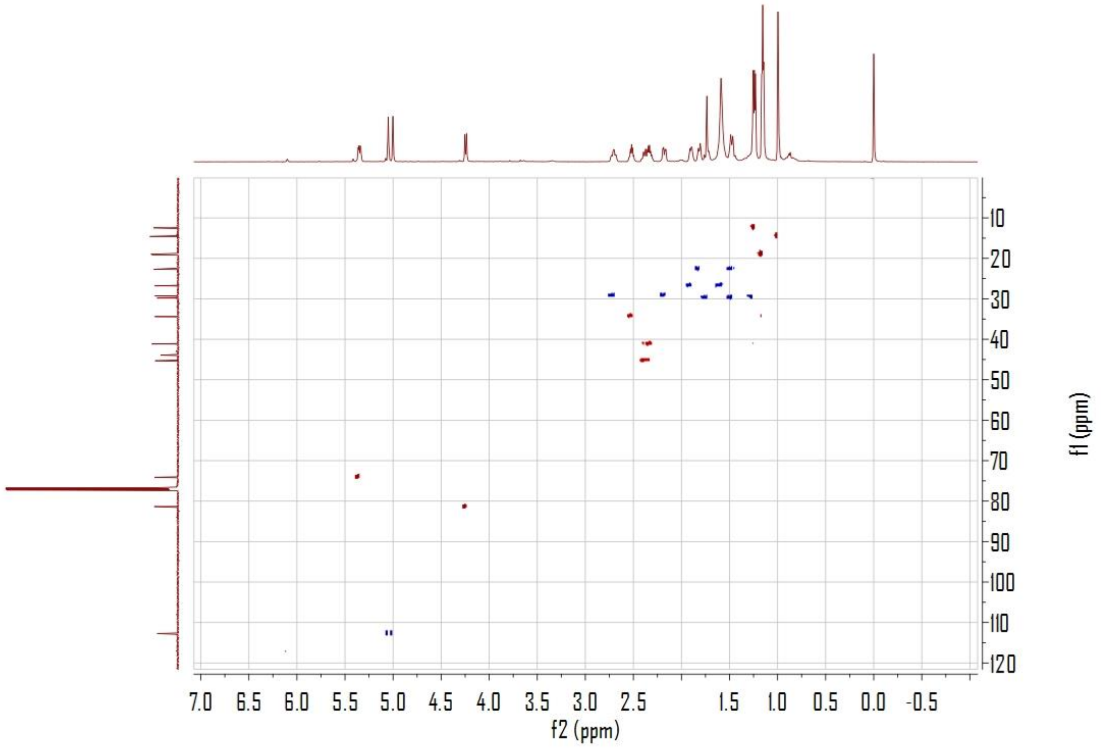

Figure S18. HSQC spectrum (CDCl3) of compound 3

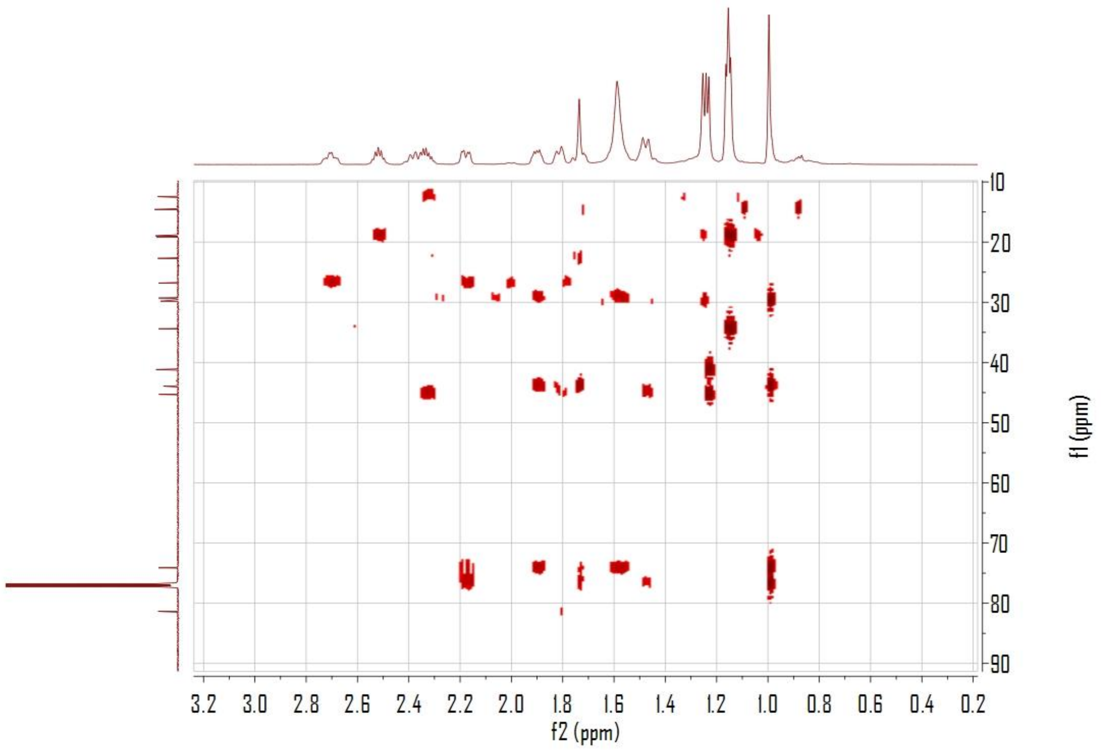

Figure S19. HMBC spectrum (CDCl3) of compound 3

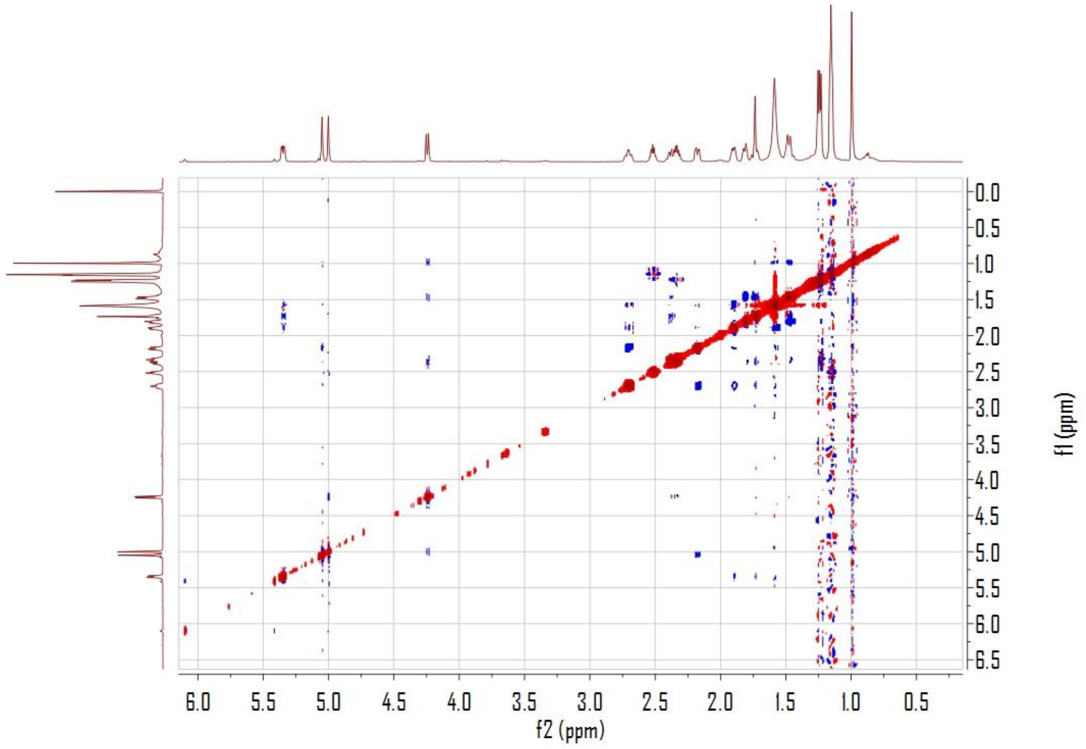

Figure S20 NOESY spectrum (CDCl3) of compound 3

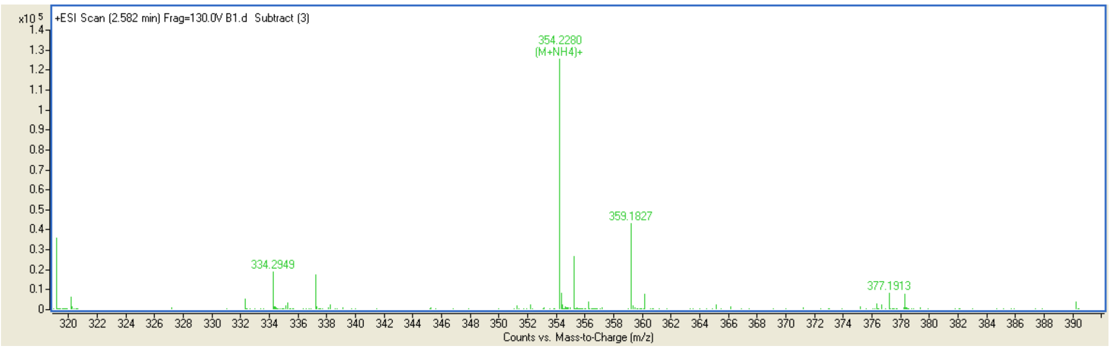

Figure S21 HRESIMS spectrum of compound 3

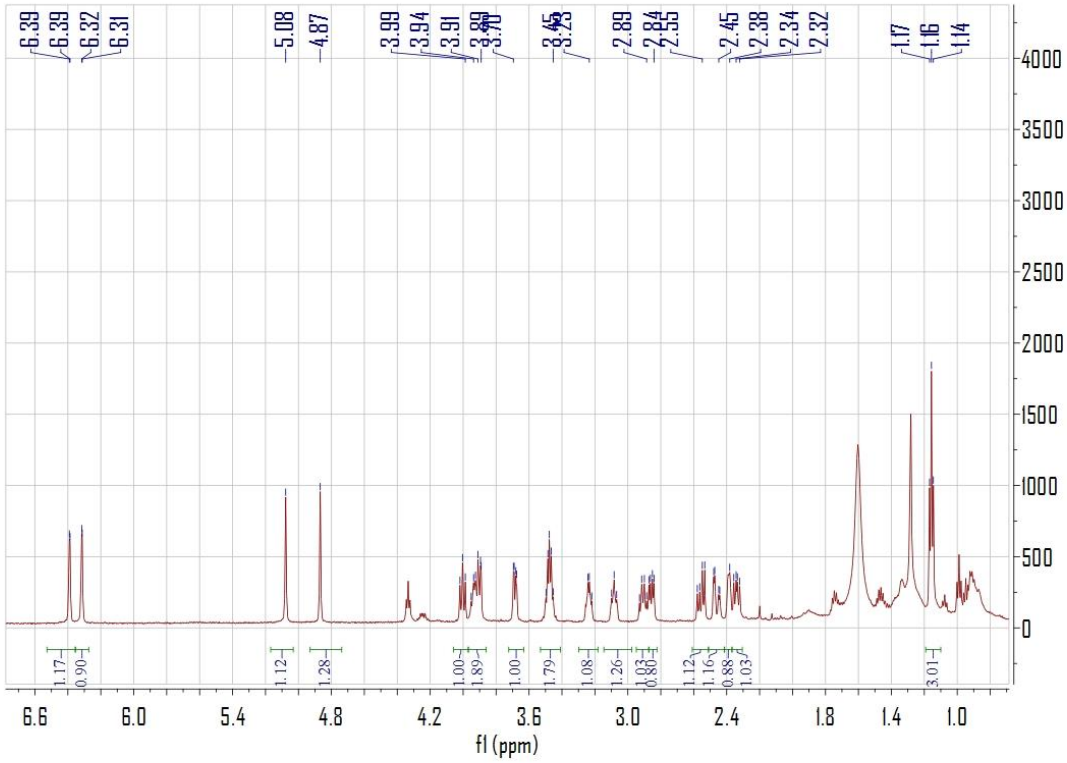

Figure S22. 1H NMR (600 MHz, CDCl3) spectrum of compound 4

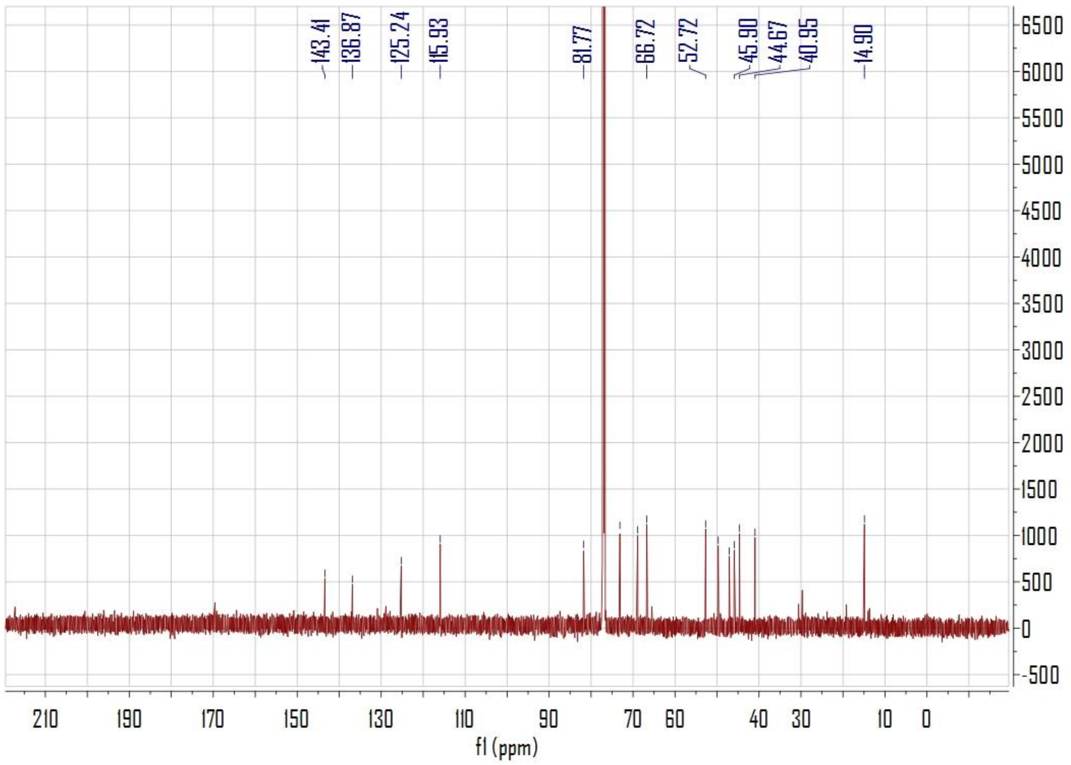

Figure S23. 13C NMR (150 MHz, CDCl3) spectrum of compound 4

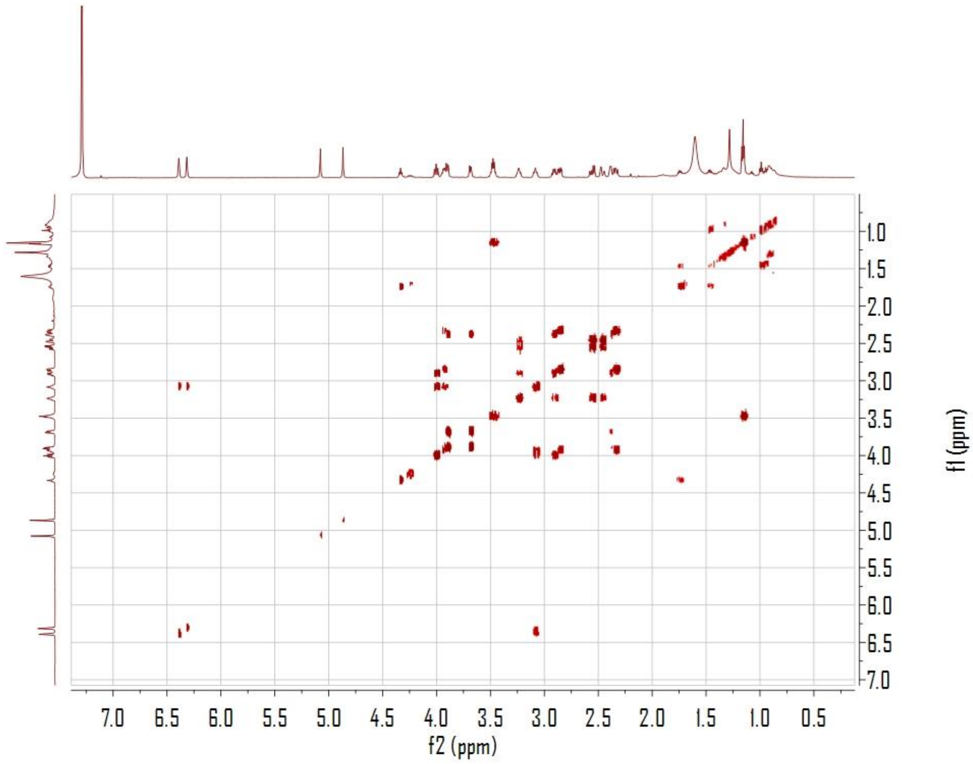

Figure S24. 1H–1H COSY spectrum (CDCl3) of compound 4

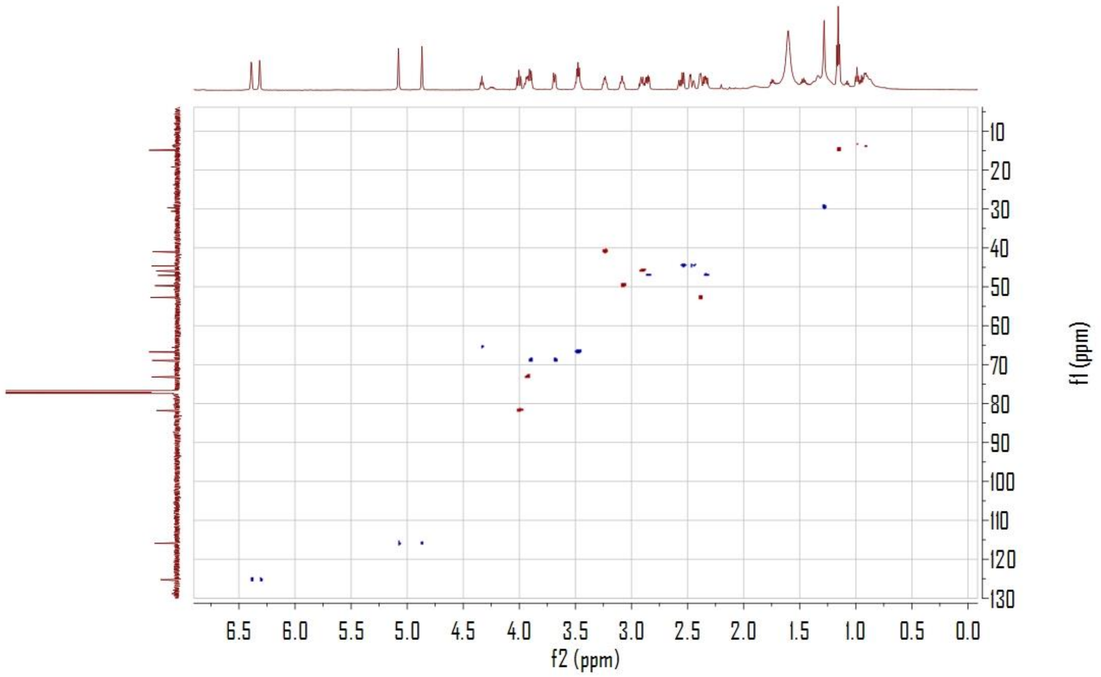

Figure S25 HSQC spectrum CDCl3) of compound 4

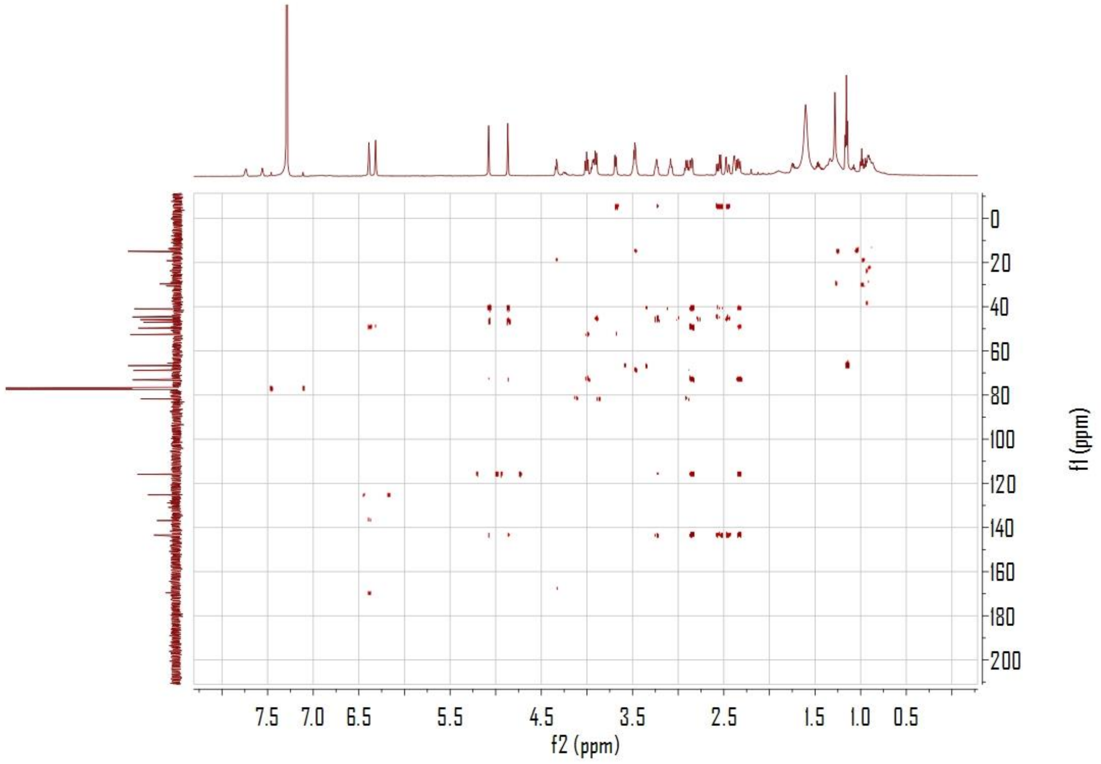

Figure S26 HMBC spectrum (CDCl3) of compound 4

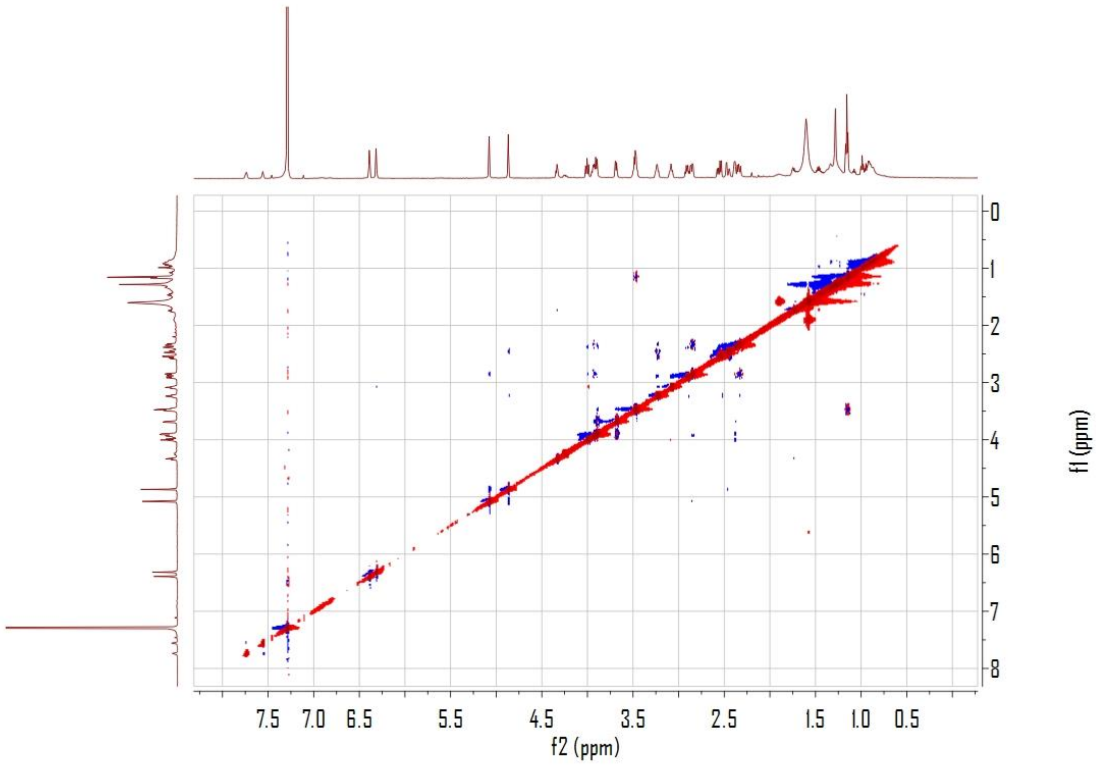

Figure S27 NOESY spectrum (CDCl3) of compound 4

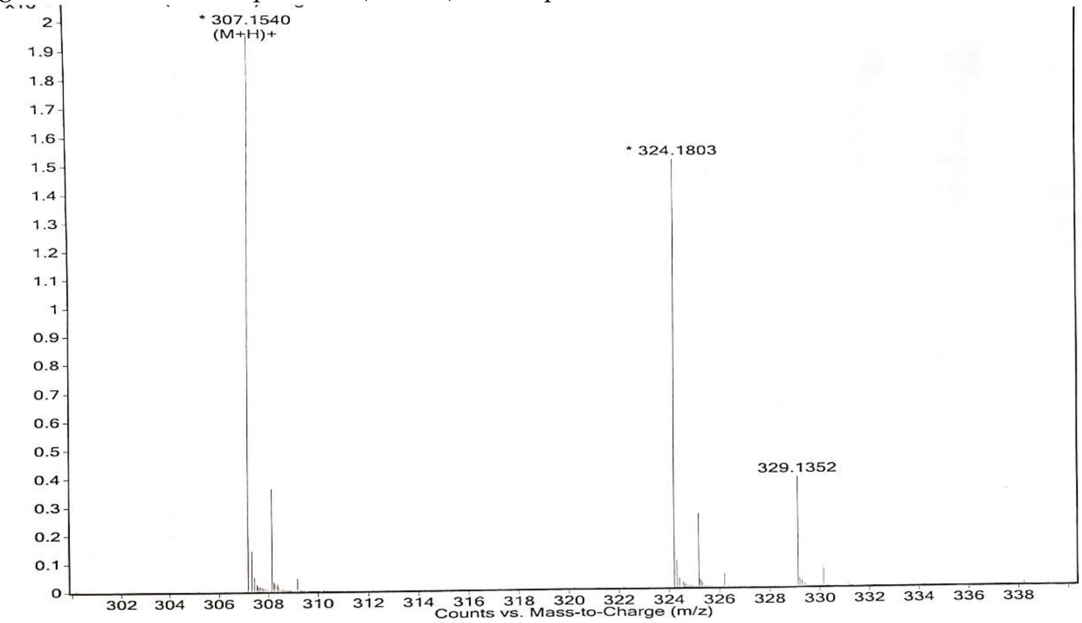

Figure S28 HRESIMS spectrum of compound 4

The ECD calculation details of compounds **1**, **3** and **4**

Systematic conformational analyses for compounds **1, 3** and **7** with the MMFF94 molecular mechanics force field were carried out using Molecular Operating Environment (MOE) ver. 2009.10. (Chemical Computing Group, Canada) software package. Subsequently, lowest energy conformers of (1R,6S,7S,10R,11S)-**1,** (1R,5R,6S,7S,10S,11S)-**3** and (1R,4R,5R,6R,7R,8S)-**4** were optimized using DFT at B3LYP/6-31G(d) basis set level using Gaussian09 package. The stationary points have been checked as the true minima of the potential energy surface by verifying they do not exhibit vibrational imaginary frequencies. The 40 lowest electronic transitions were calculated using the TDDFT methodology at the B3LYP/6-31G(d) lever. ECD spectra were stimulated using a Gaussian function with a half-bandwidth of 0.5 eV. The overall ECD spectra were then generated according to Boltzmann weighting of each conformer. The systematic errors in the prediction of the wavelength and excited-state energies are compensated for by employing UV correlation.

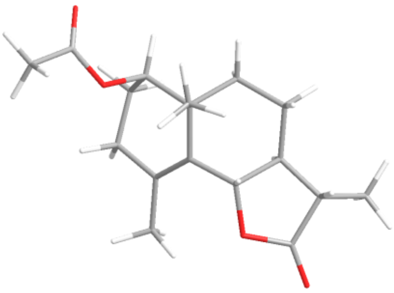	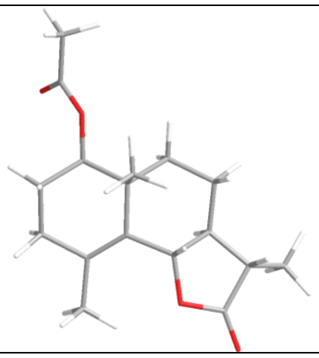
37.41 %	32.87 %
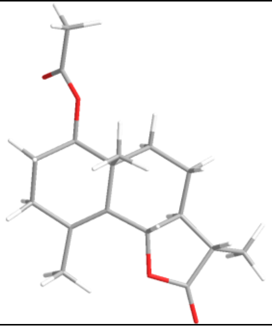	
29.72 %	

Figure S29. B3LYP/6-31G (d) optimized lowest energy conformers for (1R,6S,7S,10R,11S)-1

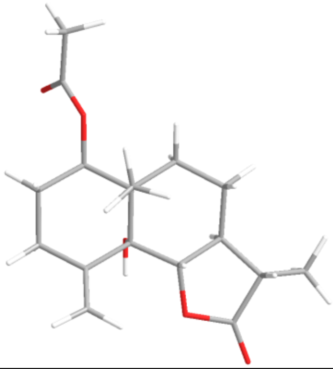	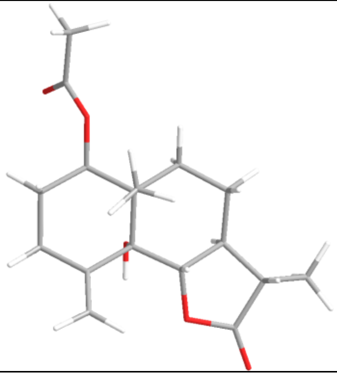
26.90 %	24.71 %

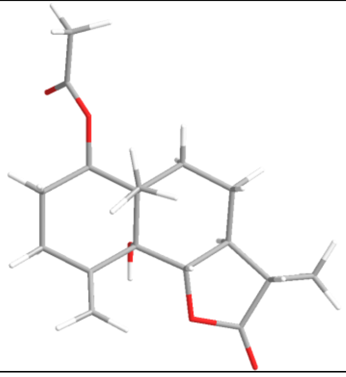	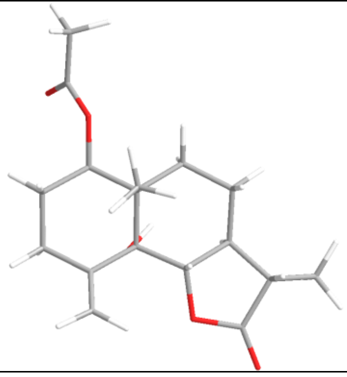
24.60 %	23.79 %

Figure S30. B3LYP/6-31G (d) optimized lowest energy conformers for (1R,5R,6S,7S,10S,11S)-3

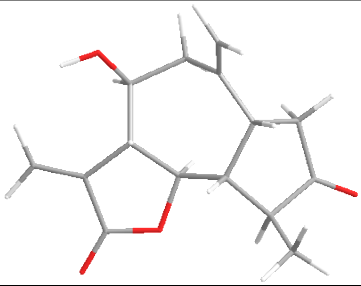	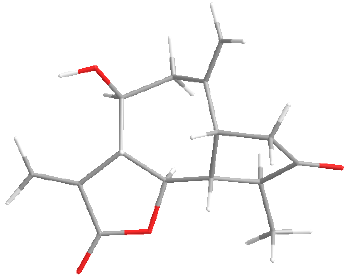
20.34%	19.37 %
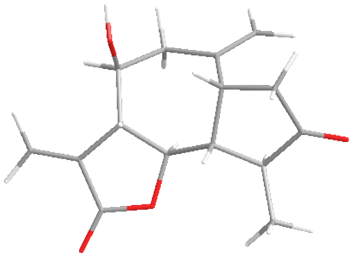	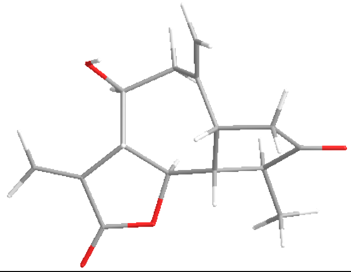
13.84 %	13.62 %
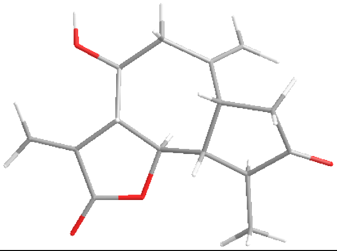	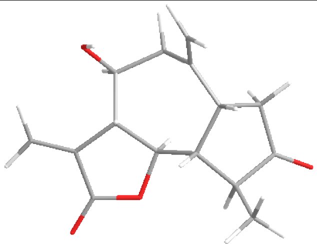
11.09 %	8.83 %
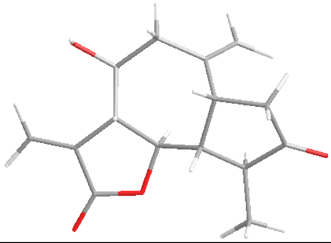	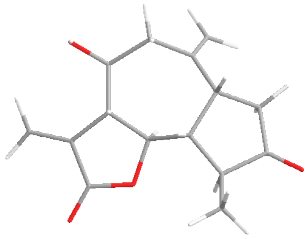
7.99 %	4.92 %

Figure S31 B3LYP/6-31G (d) optimized lowest energy conformers for (1R,4R,5R,6R,7R,8S)-4
